# Stearoyl-CoA Desaturase (SCD) Induces Cardiac Dysfunction with Cardiac Lipid Overload and Angiotensin II AT1 Receptor Protein Up-Regulation

**DOI:** 10.3390/ijms22189883

**Published:** 2021-09-13

**Authors:** Joshua Abd Alla, Yahya F. Jamous, Ursula Quitterer

**Affiliations:** 1 Molecular Pharmacology, ETH Zurich, Winterthurerstrasse 190, 8057 Zurich, Switzerland; joshua.abdalla@pharma.ethz.ch (J.A.A.); yahya.jamous@pharma.ethz.ch (Y.F.J.); 2 Institute of Pharmacology and Toxicology, University of Zurich, Winterthurerstrasse 190, 8057 Zurich, Switzerland

**Keywords:** SCD, Scd1, transgenic mice, heart failure, lipid overload, angiotensin II, AT1 receptor, AGTR1, cardiac dysfunction

## Abstract

Heart failure is a major cause of death worldwide with insufficient treatment options. In the search for pathomechanisms, we found up-regulation of an enzyme, stearoyl-CoA desaturase 1 (*Scd1*), in different experimental models of heart failure induced by advanced atherosclerosis, chronic pressure overload, and/or volume overload. Because the pathophysiological role of *Scd1*/*SCD* in heart failure is not clear, we investigated the impact of cardiac *SCD* upregulation through the generation of C57BL/6-Tg(MHCSCD)Sjaa mice with myocardium-specific expression of *SCD*. Echocardiographic examination showed that 4.9-fold-increased *SCD* levels triggered cardiac hypertrophy and symptoms of heart failure at an age of eight months. Tg-*SCD* mice had a significantly reduced left ventricular cardiac ejection fraction of 25.7 ± 2.9% compared to 54.3 ± 4.5% of non-transgenic B6 control mice. Whole-genome gene expression profiling identified up-regulated heart-failure-related genes such as resistin, adiponectin, and fatty acid synthase, and type 1 and 3 collagens. Tg-*SCD* mice were characterized by cardiac lipid accumulation with 1.6- and 1.7-fold-increased cardiac contents of saturated lipids, palmitate, and stearate, respectively. In contrast, unsaturated lipids were not changed. Together with saturated lipids, apoptosis-enhancing p53 protein contents were elevated. Imaging by autoradiography revealed that the heart-failure-promoting and membrane-spanning angiotensin II AT1 receptor protein of Tg-*SCD* hearts was significantly up-regulated. In transfected HEK cells, the expression of *SCD* increased the number of cell-surface angiotensin II AT1 receptor binding sites. In addition, increased AT1 receptor protein levels were detected by fluorescence spectroscopy of fluorescent protein-labeled AT1 receptor-Cerulean. Taken together, we found that *SCD* promotes cardiac dysfunction with overload of cardiotoxic saturated lipids and up-regulation of the heart-failure-promoting AT1 receptor protein.

## 1. Introduction

With the aging of global society, the incidence of heart failure is on the rise world-wide [1]. Heart failure is a condition where the heart function is insufficient to meet the oxygen demands of the body and its vital organs. Frequent causes of heart failure are major cardiovascular pathologies such as untreated hypertension, chronic atherosclerotic vascular disease with ensuing myocardial infarction, and chronic volume and/or pressure overload [2,3].

Several evidence-based treatment options of heart failure are available. Recommended treatments of heart failure reduce the morbidity of heart failure patients and extend life expectancy [2,3,4]. A mainstay treatment of heart failure is the inhibition of the overactive renin–angiotensin–aldosterone system (RAAS) [2,4]. The major heart-failure-promoting receptor of the RAAS is the AT1 receptor for angiotensin II, *AGTR1* [2,4]. The AGTR1-activating angiotensin II is mainly generated by the angiotensin-converting enzyme, ACE. Consequently, recommended treatment modalities of heart failure include an inhibitor of the angiotensin converting enzyme (ACE) or an angiotensin II AT1 receptor blocker, ARB [4]. Despite several prognosis-improving therapies, treatment options of heart failure are still insufficient, and diagnosis of overt heart failure is associated with a worse prognosis than that of most malignant diseases [5]. Therefore, research on pathomechanisms is urgently needed to identify new targets for treatment. Based on available concepts, previously unrecognized pathomechanisms are expected to synergize with the over-activated RAAS and other neurohumoral systems of heart failure.

To investigate the pathomechanisms of heart failure, we performed cardiac whole-genome microarray gene expression analysis of different experimental heart failure models applying the Mouse Genome MG430 2.0 array with more than 45,000 probe sets. Differentially expressed genes were classified by gene ontology (GO) analysis. GO analysis used our data of four murine experimental models, in which heart failure was triggered by major cardiovascular pathologies, such as atherosclerosis, chronic pressure, and/or volume overload [6,7]. As a model of atherosclerosis-induced heart failure, we applied aged hypercholesterolemic apolipoprotein E-deficient (*Apoe*-/-) mice with high atherosclerotic plaque load [6,7]. In a second model, symptoms of heart failure were induced in young hypercholesterolemic *Apoe*-/- mice by chronic pressure overload imposed by abdominal aortic constriction (AAC) before overt atherosclerotic plaque accumulation [6,7]. The third model induced symptoms of heart failure in *Apoe*-/- mice by chronic stimulation of the heart-failure-promoting and adipogenic transcription factor, peroxisome proliferator-activated receptor gamma (*Pparg*) with the *Pparg* agonist, rosiglitazone [6]. In the fourth model, heart failure symptoms were triggered in non-transgenic B6 mice with normal plasma cholesterol by long-term AAC-induced chronic pressure overload [6,7]. In agreement with previous whole-genome gene expression studies [8,9], data analysis of our microarray gene expression profiles identified differentially expressed groups of genes related to heart failure such as genes encoding connective tissue and cytoskeletal proteins, oxidation enzymes, and genes of cell energy processes. In addition to those previously recognized heart-failure-related gene groups, we found the consistent up-regulation of the cardiac lipid metabolic process in the four different experimental heart failure models. Among several lipid-synthesizing enzymes, there was the prominent up-regulation of the stearoyl-CoA desaturase 1 (*Scd1*) in failing hearts.

Several lines of evidence suggest a pathological role of *SCD*, notably in obesity, diabetes, and metabolic diseases [10,11]. Despite progress, the role of cardiac *Scd1*-*SCD* up-regulation in heart failure is not clear. On one hand, *Scd1* is reported to be beneficial because it protects against saturated fatty acid-induced apoptosis-enhancing catabolism [12,13,14]. On the other hand, *Scd1* seems to be detrimental for the heart because systemic *Scd1* deficiency could improve the impaired cardiac function of obese (ob/ob) mice [15,16]. In agreement with a negative impact on heart function, increased plasma contents of unsaturated palmitoleic acid as a marker of increased systemic stearoyl-CoA desaturase activity were found to be associated with an elevated risk of heart failure in human subjects [17]. Unsaturated palmitoleic acid is a major lipid product of *SCD*, which is not only associated with heart failure but also with other cardiovascular risk factors such as high blood pressure, inflammation, diabetes, and acute myocardial infarction [18,19,20,21,22]. Because knockout or inhibition of *SCD* leads to decreased levels of cardiotoxic free fatty acids, triglycerides, and ceramide [23,24], an increased SCD activity could become detrimental for the heart by increasing unsaturated and saturated lipids.

However, previous studies mainly addressed systemic functions of *SCD*, which is predominantly expressed in fat tissue and liver. By high expression in fat and liver, *SCD* accounts for systemic alterations of plasma lipid contents. In contrast, the specific impact of an increased expression of *SCD* in the heart is not known. An increase in cardiac *SCD* could be relevant because the specific increase in SCD activity in the heart could counteract saturated fatty-acid-induced worsening of left ventricular diastolic dysfunction [25]. Moreover, lipid-synthesizing enzymes such as Scd1 in atrial cardiomyocytes could promote cardiomyocyte survival under stress conditions [26].

In view of the unresolved function of *SCD* in the heart, we generated transgenic C57BL/6-Tg(MHCSCD)Sjaa (Tg-*SCD*) mice with myocardium-specific expression of *SCD*. Our study aimed to investigate the function of *SCD* up-regulation in the heart. The outcome of the study could identify *SCD* as a novel target involved in cardiac pathology and heart failure. Phenotyping of Tg-*SCD* mice revealed that the increased expression of *SCD* in the heart is a sufficient cause of cardiac dysfunction with a reduced left ventricular cardiac ejection fraction and cardiac hypertrophy at an age of 8 months. Concomitantly, *SCD* induced the accumulation of saturated lipids and the heart-failure-promoting AT1 receptor protein in the heart.

## 2. Results

### 2.1. Cardiovascular Risk Factors Trigger Up-Regulation of the Cardiac Lipid Metabolic Process in Hypercholesterolemic Apoe-/- Mice

**Figure 1:**
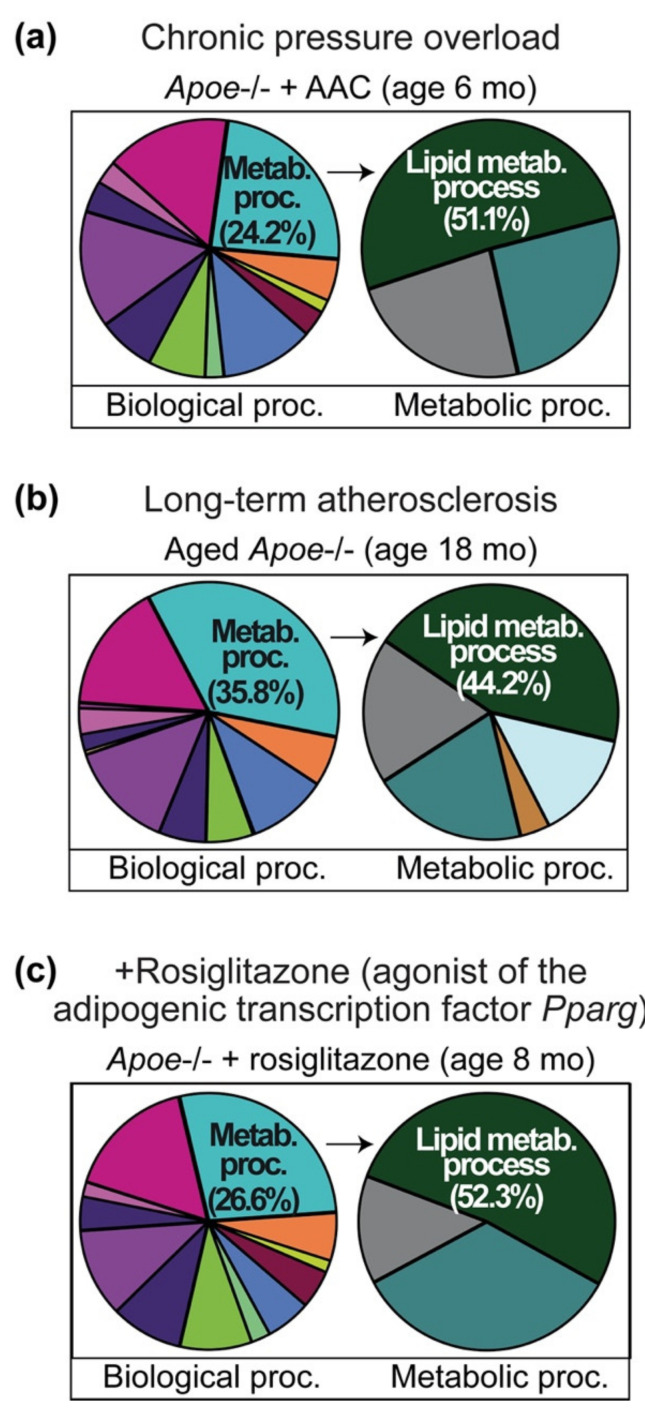
Cardiovascular pathologies trigger up-regulation of enzymes of the cardiac lipid metabolic process in hypercholesterolemic *Apoe*-/- mice. (**a**) GO analysis of up-regulated cardiac transcripts of 6-month-old *Apoe*-/- mice with 2 months of chronic pressure overload imposed by AAC compared to age-matched, sham-operated, 6-month-old *Apoe*-/- mice; (**b**) GO analysis results of aged, 18-month-old *Apoe*-/- mice with long-term atherosclerosis compared to age-matched, non-transgenic B6 hearts; (**c**) GO analysis results of 8-month-old *Apoe*-/- mice with two months of treatment with the heart-failure-promoting *Pparg* agonist, rosiglitazone, compared to untreated, 8-month-old *Apoe*-/- mice. Probe sets with significantly different signal intensities compared to the respective control group (*p* < 0.01; ≥2.0-fold difference; call present and/or intensity ≥100) were subjected to classification by GO analysis. Color codes mark different groups of genes according to GO classification. GO terms are shown in Figure 2.

To investigate pathomechanisms of heart failure, we performed gene ontology (GO) analysis of whole-genome gene expression data of three different experimental heart failure models [6,7]. In these murine models, heart failure symptoms were triggered by major cardiovascular pathologies, i.e., advanced atherosclerosis, chronic pressure overload, and/or volume overload [6,7]. GO analysis was performed of transcripts, which were significantly up-regulated (≥2-fold, *p* < 0.01) compared to the respective control group. Cardiac whole-genome gene expression data of the following heart failure models were used for GO analysis [6,7]: (I) six-month-old apolipoprotein E-deficient (*Apoe*-/-) mice with heart failure induced by two months of chronic pressure overload imposed by abdominal aortic constriction, AAC (Figure 1a); (II) aged (18-month-old) *Apoe*-/- mice with symptoms of heart failure triggered by advanced atherosclerosis and ensuing atherosclerotic narrowing of the aorta (Figure 1b); and (III) eight-month-old *Apoe*-/- mice with volume overload imposed by treatment for two months with the heart-failure-promoting peroxisome proliferator-activated receptor-gamma (*Pparg*) agonist, rosiglitazone (Figure 1c).

Among several groups of differentially expressed and heart-failure-related gene groups, GO analysis identified the prominently up-regulated cardiac “lipid metabolic process” in three different heart failure models (Figure 1a–c, and Tables S1–S3). In the GO category “primary metabolic processes”, the up-regulated cardiac “lipid metabolic process” was the predominant GO term of all three experimental heart failure models (Figure 1a–c). In this category of “primary metabolic processes”, the “lipid metabolic process” encompassed 51.1% of up-regulated probe sets of 6-month-old *Apoe*-/- mice with AAC-induced heart failure (Figure 1a). The “lipid metabolic process” comprised 44.2% of up-regulated probe sets of 18-month-old *Apoe*-/- mice with advanced atherosclerosis-induced heart failure (Figure 1b) and 52.3% of up-regulated probe sets of *Apoe*-/- mice with rosiglitazone-induced heart failure (Figure 1c).

These findings show that heart failure symptoms of hypercholesterolemic *Apoe*-/- mice, which are triggered by (I)) chronic pressure overload, (II) advanced atherosclerosis, and (III) *Pparg* activation with rosiglitazone, are accompanied by induction of enzymes of the cardiac lipid metabolic process.

### 2.2. Up-Regulation of the Cardiac Lipid Metabolic Process of Non-Transgenic B6 Mice with Heart Failure Induced by Chronic Pressure Overload

**Figure 2:**
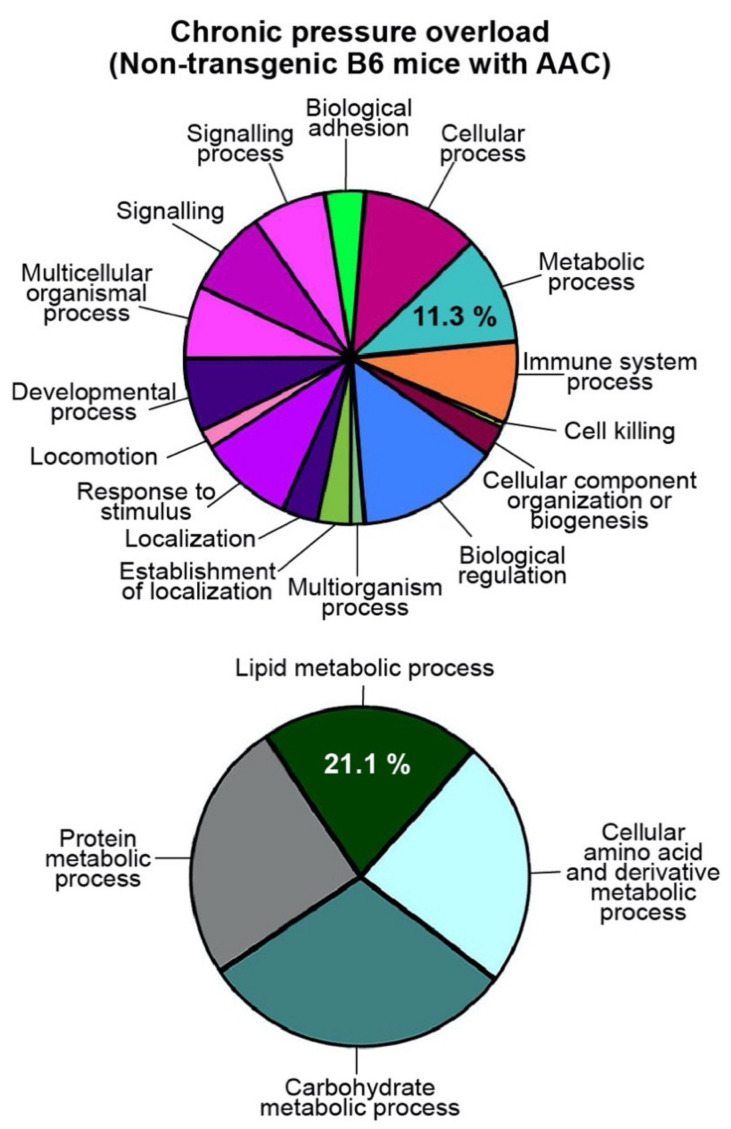
Up-regulation of the cardiac lipid metabolic process of non-transgenic B6 mice with heart failure induced by chronic pressure overload. GO analysis of up-regulated cardiac transcripts of 10-month-old, male B6 mice with 6 months of chronic pressure overload imposed by AAC compared to sham-operated, 10-month-old, male B6 hearts. Probe sets with significantly different signal intensities compared to sham-operated control group (*p* < 0.01; ≥2.0-fold difference; call present and/or intensity ≥ 100) were subjected to classification by GO analysis. GO terms are indicated.

We asked whether the lipid metabolic process was also up-regulated in a non-transgenic heart failure model without hypercholesterolemia. As an experimental heart failure model, we used 10-month-old, non-transgenic C57BL/6J (B6) mice with symptoms of heart failure induced by 6 months of abdominal aortic constriction, AAC [6,7]. GO analysis of whole-genome microarray gene expression data included significantly up-regulated transcripts (*p* < 0.01; ≥2-fold up-regulation) of B6 mice with heart failure induced by AAC compared to age-matched, sham-operated, control B6 mice (Figure 2 and Table S4).

GO analysis found that the cardiac lipid metabolic process was also up-regulated in non-transgenic B6 mice with heart failure induced by AAC (Figure 2). Up-regulated transcripts of the “lipid metabolic process” comprised 21.1% of heart-failure-induced transcripts in the category “primary metabolic processes”.

### 2.3. Concordantly Up-Regulated Genes of the Cardiac Lipid Metabolic Process in Different Heart Failure Models

**Figure 3:**
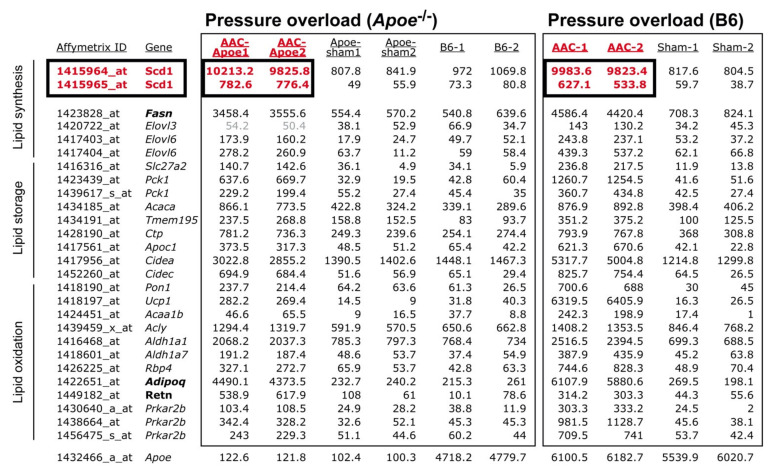
Up-regulation of the cardiac lipid metabolic process by chronic pressure overload in hypercholesterolemic *Apoe-/-* mice and non-transgenic B6 mice. DNA microarray (MG430 2.0 Array, Affymetrix) probe set intensities are shown of hearts from 6-month-old *Apoe-/-* mice with 2 months of pressure overload imposed by AAC (AAC-Apoe) compared to age-matched, sham-operated *Apoe-/-* hearts (Apoe-sham) and age-matched, non-transgenic B6 controls (B6). The right panel shows probe set intensities of 10-month-old B6 mice with six months of AAC-induced pressure overload (AAC) compared to age-matched, sham-operated, non-transgenic B6 controls (Sham). Data of two gene chips per group with cRNAs from four hearts per gene chip are shown. Probe sets of the “lipid metabolic process” with significantly different signal intensities compared to respective control group and concordant up-regulation in different heart failure models are listed (*p* < 0.01; ≥2.0-fold difference; call present and/or intensity ≥ 100).

**Figure A1:**
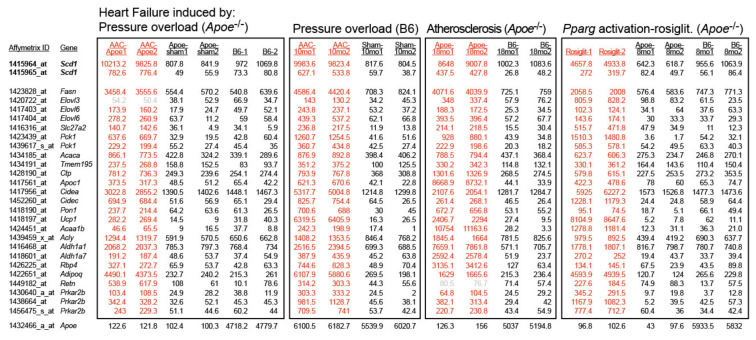
Up-regulation of the cardiac lipid metabolic process in experimental heart failure models. Heart failure was induced in 6-month-old, male *Apoe*^-/-^ mice by two months of pressure overload imposed by abdominal aortic constriction, AAC (AAC-Apoe1; AAC-Apoe2). Age-matched (6-month-old), male, sham-operated *Apoe*^-/-^ mice (Apoe-sham1; Apoe-sham2), and age-matched, non-transgenic, male B6 mice (B6-1; B6-2) were used as controls. In the second model, heart failure was induced in 10-month-old, male, non-transgenic B6 mice by pressure overload imposed by 6 months of AAC (AAC-10mo1; AAC-10mo2). Age-matched (10-month-old), sham-operated, male B6 mice (Sham-10mo1; Sham-10mo2) served as controls. In the third model, heart failure was induced by long-term atherosclerosis of 18-month-old, male *Apoe*^-/-^ mice (Apoe-18mo1; Apoe-18mo2). Age-matched, male B6 mice (B6-18mo1; B6-18mo2) were used as controls. In the fourth model, heart failure of 8-month-old, male *Apoe*^-/-^ mice was induced by *Pparg* activation with 2 months of rosiglitazone (30 mg/kg/d in drinking water) treatment (Rosiglit-1; Rosiglit-2). Age-matched (8-month-old), male *Apoe*^-/-^ mice (Apoe-8mo1; Apoe-8mo2), and B6 mice (B6-8mo1; B6-8mo2) served as controls. At the end of the observation periods, hearts were isolated and total RNA was prepared and processed for microarray gene expression profiling. Two gene chips are shown for each group (four individuals/gene chip). Intensities of probe sets of the lipid metabolic process (GO analysis) are marked in red, which were concordantly up-regulated (-fold change ≥ 2; *p* ≤ 0.01 or 0.05) in the different heart failure models compared to respective controls. As a control, the probe set detecting *Apoe* is also shown. Whole genome microarray gene expression data of all models are available at the NCBI GEO database with accession numbers GSE25765, GSE25766, GSE25767, and GSE25768.

DNA microarray probe set intensities of heart-failure-related and concordantly up-regulated genes of the cardiac lipid metabolic process of different heart failure models are listed in Figure 3 and Figure A1. Genes of the cardiac lipid metabolic process were similarly up-regulated in three different heart failure models of hypercholesterolemic *Apoe*-/- mice and in failing hearts of non-transgenic B6 mice (Figure 3 and Figure A1). Notably, probe set intensities of up-regulated transcripts of the cardiac lipid metabolic process were comparable between non-transgenic C57BL/6J (B6) mice with heart failure induced by AAC and hypercholesterolemic *Apoe*-/- mice subjected to AAC (Figure 3).

Up-regulated transcripts of the lipid metabolic process were sorted into the categories: lipid synthesis, lipid storage, and lipid oxidation (Figure 3). Several of the up-regulated genes have a documented relationship to heart failure such as the major fatty-acid-synthesizing enzyme, fatty acid synthase, *Fasn* [6,7], and the heart failure markers adiponectin, *Adipoq* [27], and resistin, *Retn* [28].

The role of several of the up-regulated lipid genes in failing hearts was not previously elucidated. Among different genes of the lipid metabolic process, *Scd1*, the stearoyl-CoA desaturase-1, showed the highest signal intensity (Figure 3) but its function in the heart is not clear. Because the effect of *Scd1* up-regulation in the heart is not known, this study aims to elucidate the consequences of increased *Scd1-SCD* expression levels on cardiac phenotype and heart function.

### 2.4. Generation of Tg-SCD Mice with Myocardium-Specific SCD Expression

**Figure 4:**
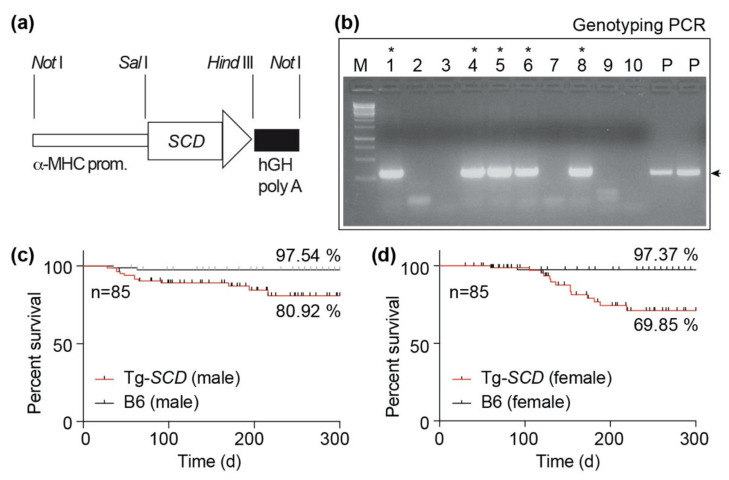
Generation of Tg-*SCD* mice with myocardium-specific expression of *SCD*. (**a**) Scheme of plasmid used for generation of Tg-*SCD* mice; (**b**) identification of mice with stable genomic insertion of the *SCD* transgene by genotyping PCR. *SCD*-transgenic mice are marked with a star (*), (P: plasmid control); (**c**) survival rate of male Tg-*SCD* mice compared to male, non-transgenic B6 mice; (**d**) survival analysis of female Tg-*SCD* mice compared to female, non-transgenic B6 mice (*n* = 85 mice per group).

Cardiac *Scd1* was found to be up-regulated in different models of heart failure and cardiac biopsies of patients with heart failure [6,7]. However, the impact of cardiac *Scd1*-*SCD* up-regulation is unknown. *Scd1* up-regulation was reported to protect the heart against lipid-induced cardiac damage [12]. In contrast, another study found that *Scd1* could be deleterious for the heart because *Scd1* deficiency improved the cardiac phenotype of obese (ob/ob) mice [15,16]. In view of these divergent data, we investigated the phenotype of cardiac *SCD* up-regulation by generation of transgenic mice with myocardium-specific *SCD* expression under the control of the heart-muscle-specific α-MHC-promoter (Figure 4a). There is no complication on α-MHC-promoter-driven expression of *SCD* expected, which could be related to, e.g., hypercholesterolemia or hemodynamic intervention, because the *SCD* transgene was expressed in non-transgenic B6 mice without hypercholesterolemia and without hemodynamic intervention. Transgenic mice were generated by the injection of linearized plasmid DNA into the pronucleus of embryos, followed by oviduct transfer of two-cell stage embryos into the oviducts of foster mice. After weaning, genomic DNA was isolated from biopsies, and transgenic mice were identified by genotyping PCR with DNA oligonucleotides, which specifically amplify the DNA of the *MHC-SCD* transgene (Figure 4b). Oligonucleotides are specific for the transgenic *MHC-SCD* DNA, and do not cross-react with the endogenous murine *Scd1* gene. Founder mice were used for further breeding to generate C57BL/6-Tg(MHCSCD) mice.

Survival analysis showed a decreased survival rate of male Tg-*SCD* mice of 80.92% compared to the survival rate of 97.54% of non-transgenic, male B6 controls during the observation period of 10 months (Figure 4c). Female C57BL/6-Tg(MHCSCD) mice had a decreased survival rate of 69.85% (Figure 4d).

### 2.5. Immunohistological Analysis Shows Cardiac Enlargement of Tg-SCD Mice and Increased Cardiac SCD Protein Levels

**Figure 5:**
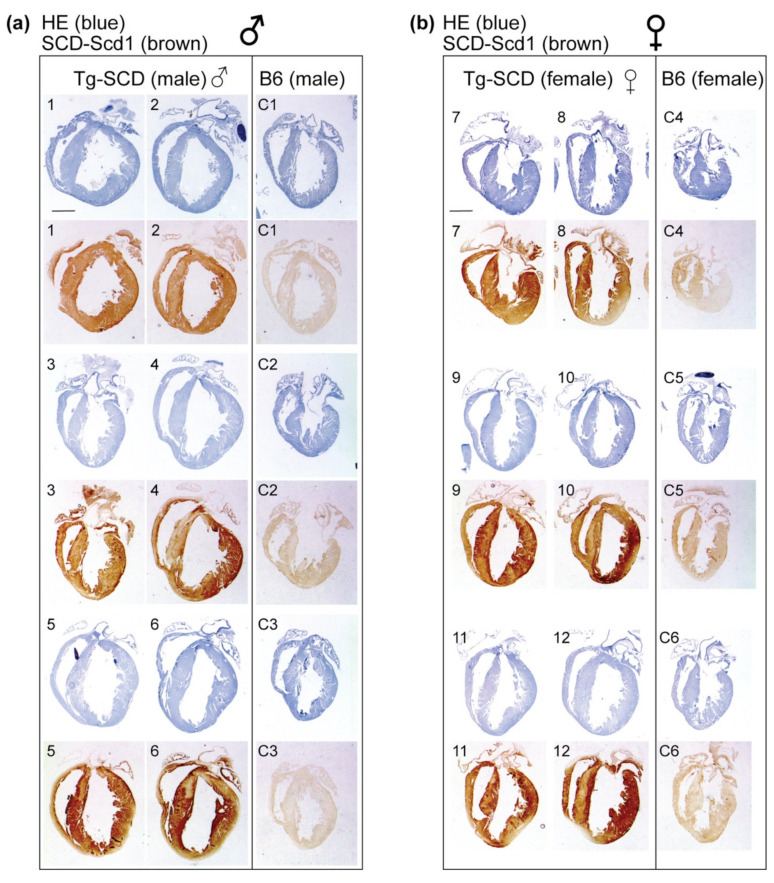
Immunohistological analysis shows cardiac enlargement of Tg-*SCD* mice and increased cardiac SCD protein levels. (**a**,**b**) Hematoxylin-eosin-stained heart sections (HE blue) and immunohistological staining with knockout-validated SCD-specific antibodies (SCD-Scd1 brown) of increased myocardial SCD protein levels on cardiac specimens of eight-month-old male (**a**) and female (**b**) Tg-*SCD* mice compared to those of non-transgenic B6 controls (bar: 2 mm; *n* = 6 male (1–6) and *n* = 6 female (7–12) Tg-*SCD* mice; *n* = 3 male (C1–C3) and *n* = 3 female (C4–C6) B6 control mice). Quantitative data are shown in Figure A2 in Appendix A.

In view of the increased mortality of Tg-*SCD* mice, the cardiac phenotype of Tg-*SCD* mice was determined by histological analysis of cardiac specimens of 8-month-old, male and female Tg-*SCD* mice. At an age of 8 months, hearts from Tg-*SCD* mice were isolated and characterized by histology. Histological analysis was performed with sections of paraffin-embedded hearts from 8-month-old, male and female Tg-*SCD* mice compared to age-matched, non-transgenic, male and female B6 mice (Figure 5a,b). Hematoxylin-eosin (HE) staining showed enlargement of cardiac ventricles with thickened myocardium indicative of cardiac hypertrophy of Tg-*SCD* hearts (Figure 5a,b).

**Figure A2:**
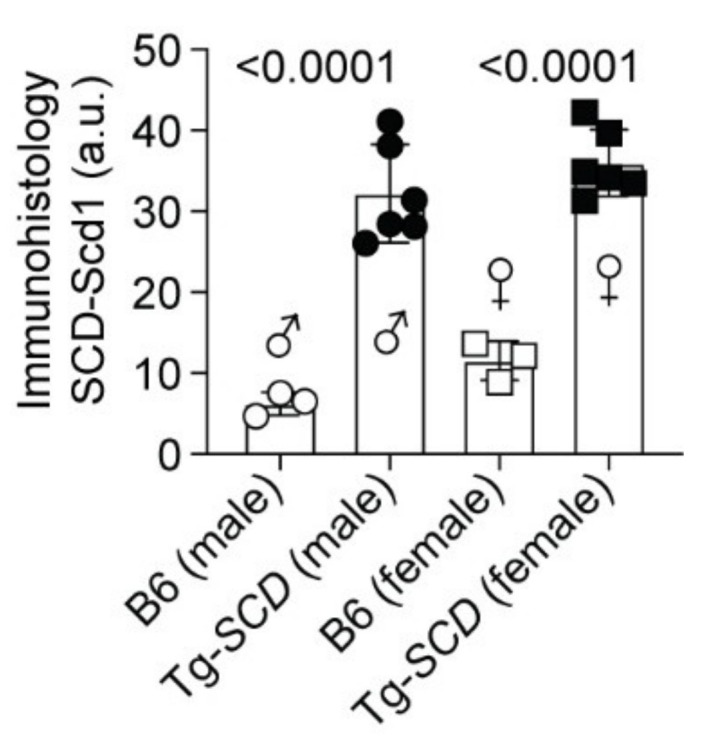
Quantitative evaluation of immunohistological detection of SCD-Scd1 protein by knockout-validated SCD-specific antibodies on hearts of male and female Tg-*SCD* mice and non-transgenic B6 controls (±s.d.; *n* = 6 male and female Tg-*SCD* mice; *n* = 3 male and female B6 mice; ANOVA and Tukey’s test). All immunohistological images are shown in Figure 5.

Immunohistology was performed to detect cardiac SCD-Scd1 protein contents of Tg-*SCD* mice and non-transgenic B6 controls. The cardiac SCD-Scd1 protein was determined by immunohistology with knockout-validated SCD-Scd1-specific antibodies. Immunohistological analysis revealed significantly increased cardiac SCD-Scd1 protein contents of 8-month-old male and female Tg-*SCD* mice with cardiac hypertrophy compared to age-matched, non-transgenic B6 mice (Figure 5a,b and Figure A2). Quantitative evaluation of immunohistological data showed 5.16 ± 0.96-fold and 3.11 ± 0.36-fold increased cardiac SCD-Scd1 levels of male and female Tg-*SCD* mice, respectively, compared to non-transgenic B6 mice (Figure A2). Cardiac SCD-Scd1 protein contents were not significantly different between male and female Tg-*SCD* mice (Figure A2).

### 2.6. Immunoblot Analysis Shows Increased Cardiac SCD and Pro-Apoptotic p53 Protein Levels of Tg-SCD Mice

**Figure 6:**
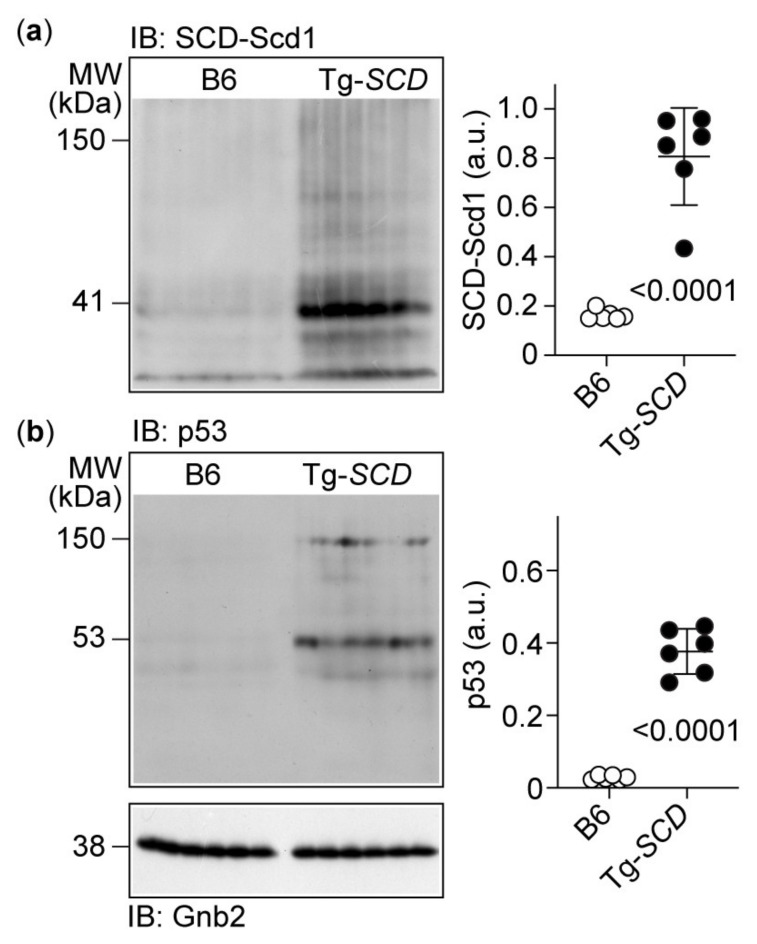
Immunoblot analysis shows increased cardiac SCD and pro-apoptotic p53 protein levels of Tg-*SCD* mice. (**a**) Immunoblot detection (IB) of SCD-Scd1 was performed in cardiac lysates of 8-month-old Tg-*SCD* mice and compared to age-matched, non-transgenic B6 mice (left panel). The right panel shows quantitative data (*n* = 6 mice per group); (**b**) immunoblot detection of p53 (IB: p53) with p53-specific antibodies in lysates of Tg-*SCD* hearts compared to non-transgenic B6 mice (left panel), and quantitative immunoblot data (right panel; *n* = 6 mice per group). The lower immunoblot detects Gnb2 as a loading control. *P* values are indicated and were determined by the unpaired, two-tailed *t*-test.

To further analyze cardiac SCD-Scd1 protein levels of Tg-*SCD* mice, immunoblot analysis was performed (Figure 6).

At an age of 8 months, hearts from Tg-*SCD* mice were isolated and cardiac SCD-Scd1 protein levels were quantified by immunoblot with knockout-validated SCD-Scd1-specific antibodies. Quantitative evaluation of western blot images showed the significantly 4.9-fold-increased cardiac SCD-Scd1 protein levels of Tg-*SCD* mice compared to those of non-transgenic B6 mice (Figure 6a).

Concomitantly with increased cardiac SCD-Scd1 protein levels, hearts from Tg-*SCD* mice showed increased levels of the apoptosis-enhancing protein, p53 (Figure 6b). The immunoblot detected the predominant monomeric p53 together with a partially aggregated p53 form at 150 kDa. Taken together, high cardiac SCD-Scd1 protein levels of Tg-*SCD* hearts are accompanied by an increase in the pro-apoptotic protein p53. The induction of myocardial p53 in Tg-*SCD* mice could be indicative of heart failure because failing myocardium shows increased p53 protein contents [29].

### 2.7. Tg-SCD Mice Have a Heart Failure Phenotype with Cardiac Hypertrophy and Cardiac Dysfunction

**Figure 7:**
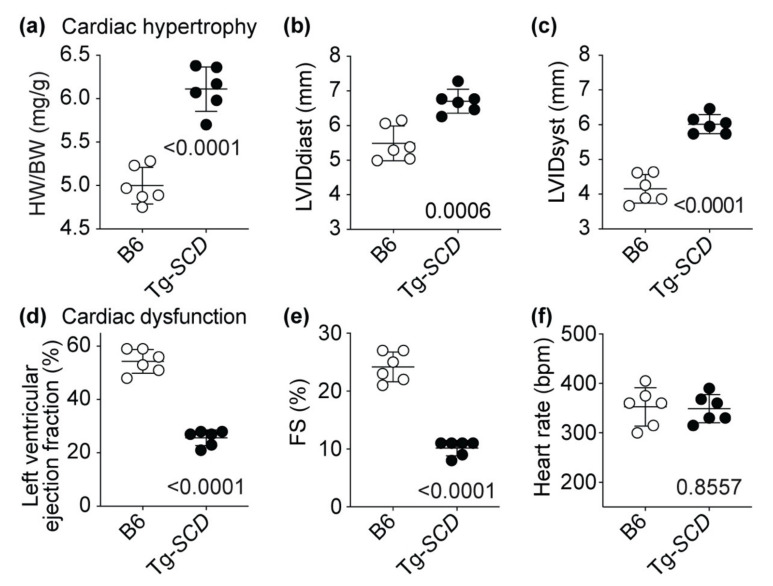
Tg-*SCD* mice have a heart failure phenotype with cardiac hypertrophy and cardiac dysfunction. (**a**) Increased heart-weight to body-weight ratio (HW/BW) of 8-month-old, male Tg-*SCD* mice compared to age-matched, non-transgenic, male B6 mice; (**b**) echocardiography detected an increased LVIDdiast of Tg-*SCD* mice; (**c**) the LVIDsyst of Tg-*SCD* mice was also higher compared to non-transgenic B6 mice; (**d**) cardiac dysfunction of Tg-*SCD* mice was reflected by a reduced left ventricular cardiac ejection fraction; (**e**) reduced fractional shortening (FS) of Tg-*SCD* mice; (**f**) heart rates of Tg-*SCD* mice were not significantly different from those of non-transgenic controls (±s.d.; *n* = 6 mice per group; unpaired, two-tailed *t*-test).

**Figure A3:**
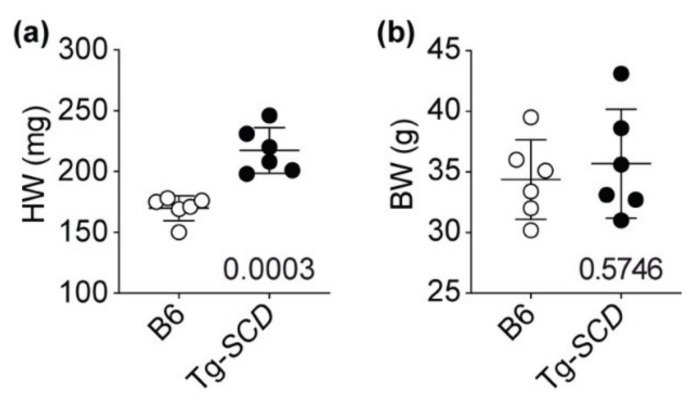
Heart weights (**a**) and body weights (**b**) of 8-month-old, male Tg-*SCD* mice compared to 8-month-old, male, non-transgenic B6 controls (±s.d.; *n* = 6 mice per group; unpaired, two-tailed *t*-test).

Assessment of heart-weight to body-weight ratio confirmed the phenotype of cardiac hypertrophy of Tg-*SCD* mice (Figure 7a). As control, body-weights of Tg-*SCD* mice were not significantly different from those of non-transgenic B6 controls (Figure A3). In agreement with cardiac enlargement, echocardiographic examination showed an increased left ventricular inner diameter at end-diastole (LVIDdiast) and end-systole (LVIDsyst) of Tg-*SCD* mice (Figure 7b,c).

Concomitantly with cardiac hypertrophy, Tg-*SCD* mice developed cardiac dysfunction at an age of 8 months (Figure 7d). Cardiac dysfunction of Tg-*SCD* mice was documented by echocardiography with a significantly reduced left ventricular cardiac ejection fraction (LVEF) of 25.7 ± 2.9% compared to 54.3 ± 4.5% of B6 controls (Figure 7d). In addition, fractional shortening (FS) of Tg-*SCD* mice was significantly decreased to 10.2 ± 1.3%, whereas the FS of B6 mice was 24.2 ± 2.6% (Figure 7e). As a control, under ketamine–medetomidine anesthesia, heart rate was not significantly different between Tg-*SCD* mice and non-transgenic B6 controls (Figure 7f). Together, these experiments show that Tg-*SCD* mice develop a phenotype of heart failure with cardiac dysfunction and cardiac hypertrophy at an age of 8 months.

### 2.8. Gene Expression Profiling of Tg-SCD Mice Shows Up-Regulation of Heart Failure-Related Lipid Genes

**Figure 8:**
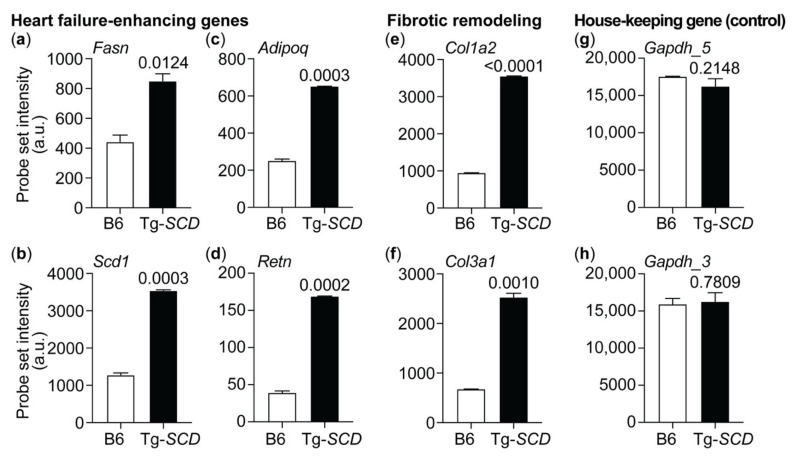
Gene expression profiling of Tg-*SCD* mice shows up-regulation of heart-failure-related lipid genes. (**a**) Up-regulation of *Fasn* (1423828_at) in Tg-*SCD* hearts; (**b**) increased intensity of probe set detecting *Scd1* (1415964_at); (**c**,**d**) up-regulation of heart-failure-related genes, *Adipoq* (1422651_at), and *Retn* (1449182_at), in Tg-*SCD* hearts; (**e**,**f**) increased intensities of probe sets detecting *Col1a2* (1423110_at) and *Col3a1* (1427884_at) are indicative of adverse fibrotic remodeling in Tg-*SCD* hearts; (**g**,**h**) comparable signal intensities of probe sets detecting *Gapdh* (AFFX-GapdhMur/M32599_5_at; AFFX-GapdhMur/M32599_3_at). Data of two gene chips per group with cRNAs from four hearts per gene chip are shown (±s.d.; *n* = 2 gene chips per group; *p* values are indicated and were determined by the unpaired, two-tailed *t*-test).

To further analyze the heart failure phenotype of Tg-*SCD* mice, we performed whole-genome microarray gene expression profiling (Figure 8).

Gene expression analysis detected up-regulated heart-failure-related genes of the lipid metabolic process (Figure 8a–d). The probe set intensity of the major palmitate-synthesizing and heart-failure-promoting *Fasn* was significantly higher in Tg-*SCD* hearts in comparison to that in non-transgenic B6 hearts (Figure 8a). In addition to *Fasn*, Tg-*SCD* hearts showed up-regulation of the murine *Scd1* transcript (Figure 8b). This finding is noteworthy because the GeneChip Mouse Genome MG430 2.0 array (Affymetrix) does not detect the human *SCD* transgene. Thus, *SCD*-induced symptoms of heart failure are accompanied by up-regulation of the murine *Scd1* transcript as another heart-failure-related gene of the lipid metabolic process. In agreement with heart failure symptoms, Tg-*SCD* hearts displayed increased expression of heart-failure-related markers, *Adipoq* and *Retn* (Figure 8c,d).

Myocardial fibrosis with increased collagen synthesis is part of the pathological cardiac remodeling process of heart failure [30]. Adverse fibrotic cardiac remodeling is attributed in part to cardiac collagen up-regulation and deposition, which contributes to increased myocardial stiffness and impaired cardiac function [30]. Concomitantly with cardiac dysfunction and heart failure, Tg-*SCD* hearts displayed the significant up-regulation of two major cardiac collagens, which are of type 1 and type 3 [30,31], i.e., *Col1a2* and *Col3a1* (Figure 8e,f). As a control, signal intensities of probe sets detecting the house-keeping gene, *Gapdh*, were not significantly different between Tg-*SCD* hearts and B6 controls (Figure 8g,h).

Taken together, gene expression analysis shows up-regulation of heart-failure-enhancing genes in Tg-*SCD* mice. This up-regulation of heart-failure-related genes complements the phenotype of heart failure with cardiac dysfunction of Tg-*SCD* mice, which was documented by echocardiography.

### 2.9. Immunoblot Detection Confirms Up-Regulation of Cardiac Fasn and Adipoq Proteins of Tg-SCD Mice

**Figure 9:**
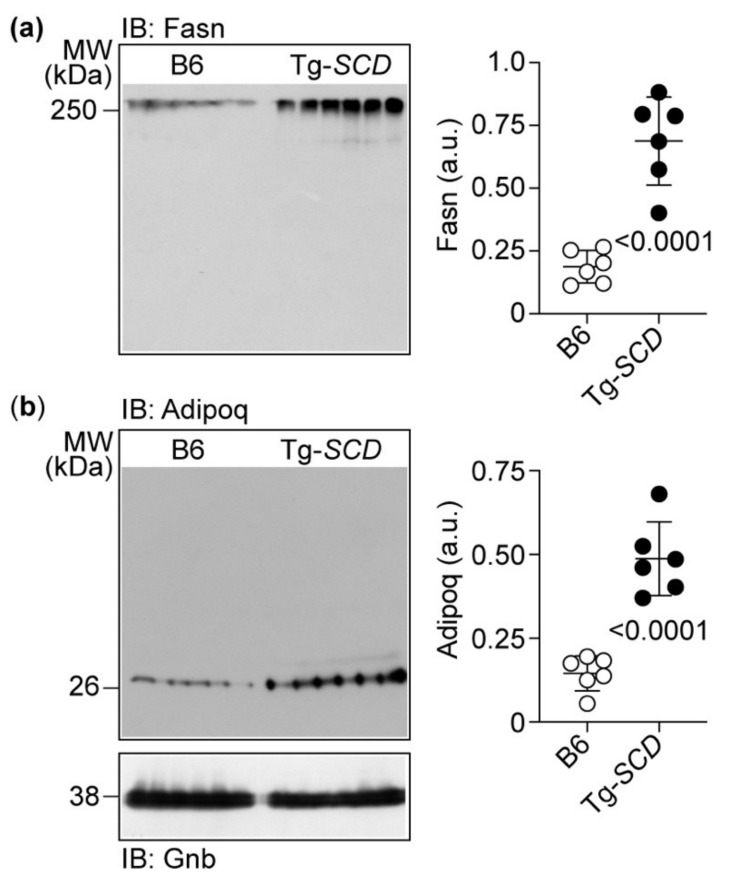
Immunoblot detection confirms up-regulation of cardiac Fasn and Adipoq in Tg-*SCD* mice. (**a**,**b**) Increased cardiac protein levels of Fasn (**a**), and Adipoq in Tg-*SCD* mice (**b**). Left panels show immunoblot images, and right panels show quantitative data (±s.d.; *n* = 6 mice per group; unpaired, two-tailed *t*-test).

Immunoblot detection was performed to validate microarray gene expression data (Figure 9).

Immunoblot analysis documented increased cardiac Fasn protein levels in Tg-*SCD* mice compared to those in non-transgenic B6 controls (Figure 9a). Cardiac Fasn protein levels of Tg-*SCD* mice were increased 3.7 ± 0.9-fold compared to those of non-transgenic B6 mice (Figure 9a).

Immunoblot detection also confirmed increased Adipoq levels of Tg-*SCD* hearts (Figure 9b). Adipoq levels of Tg-*SCD* hearts were 3.4 ± 0.8-fold higher than those of B6 hearts (Figure 9b). Together, these data show that Tg-*SCD* mice develop heart failure with cardiac hypertrophy and cardiac dysfunction at an age of 8 months with concomitantly increased cardiac protein levels of heart-failure-enhancing genes, Fasn and Adipoq.

### 2.10. Accumulation of Saturated Lipids in Tg-SCD Hearts

**Figure 10:**
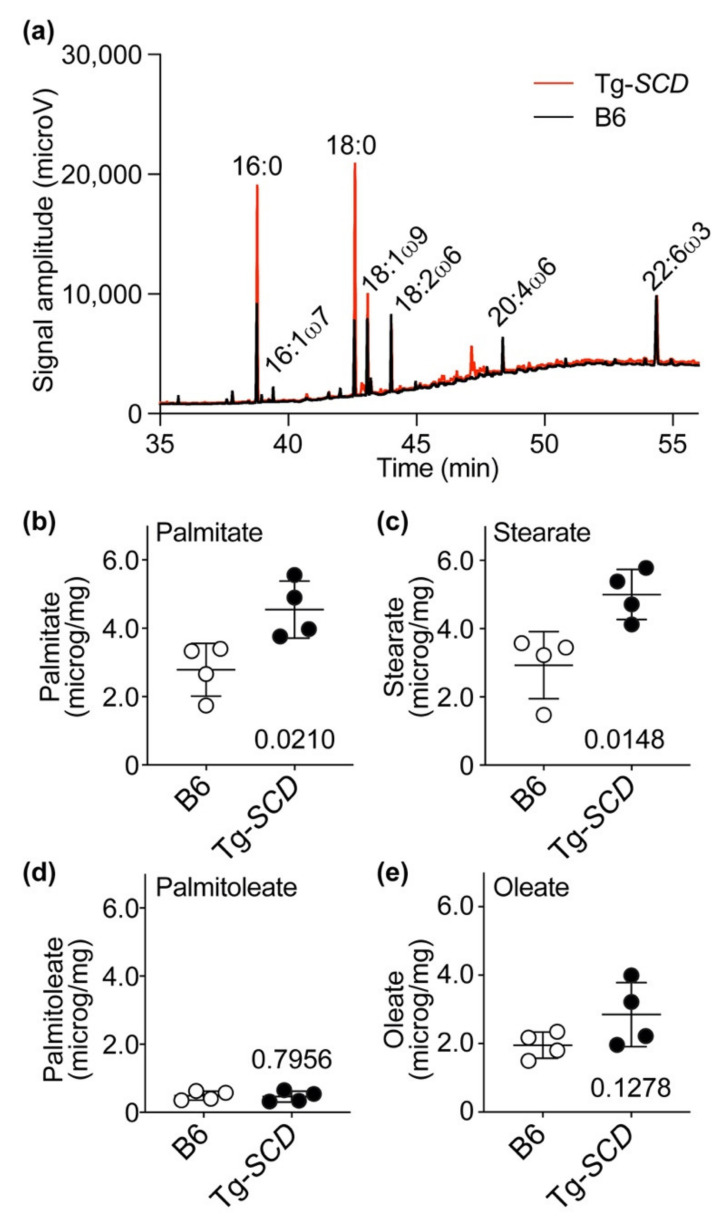
Accumulation of saturated lipids in Tg-*SCD* hearts. (**a**) Representative GC analysis of cardiac lipids of a Tg-*SCD* heart compared to that of a non-transgenic B6 control heart; (**b**) cardiac palmitate contents of 8-month-old, male Tg-*SCD* mice compared to those of age-matched, male B6 mice; (**c**) cardiac stearate contents of Tg-*SCD* mice and B6 controls; (**d**) cardiac contents of mono-unsaturated palmitoleate in Tg-*SCD* mice are not significantly different from those in B6 controls; (**e**) contents of non-saturated oleate in Tg-*SCD* hearts are not significantly different from those in B6 controls. Panel (**a**) shows a representative experiment, and panels (**b**–**e**) show quantitative data (*n* = 4 mice/group; ± s.d., unpaired, two-tailed *t*-test).

We investigated whether the up-regulated Fasn and increased SCD-Scd1 contents of Tg-*SCD* hearts led to an increased cardiac lipid load (Figure 10).

**Figure A4:**
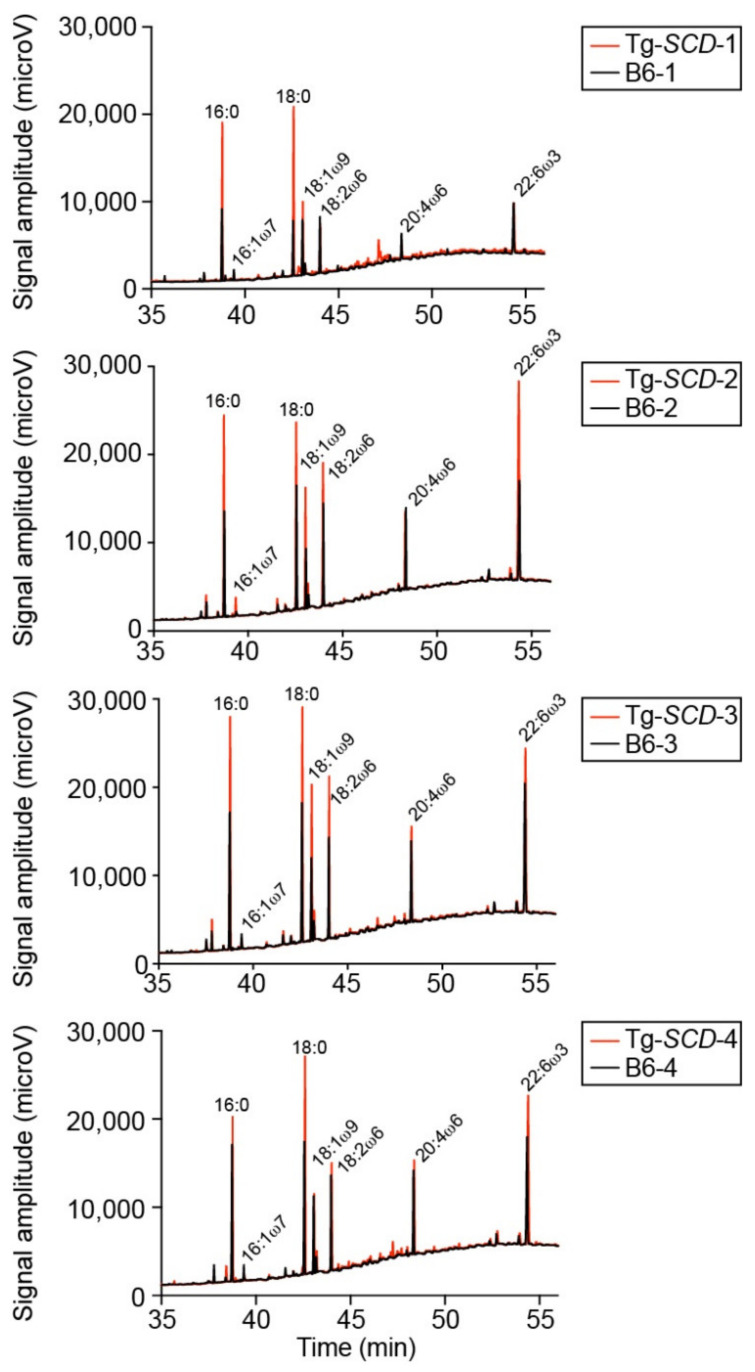
Cardiac lipid analyses of 8-month-old, male Tg-*SCD* hearts (*n* = 4) and 8-month-old, male B6 control hearts (*n* = 4). Cardiac lipids were extracted and analyzed after transesterification by GC analysis with flame ionization detection.

Cardiac lipids were analyzed after transesterification by gas chromatographic (GC) analysis with flame ionization detection (FID). For cardiac lipid analysis, we used the hearts of 8-month-old, male Tg-*SCD* mice and age-matched, male B6 control mice. Lipid analysis found that Tg-*SCD* hearts had increased levels of saturated fatty acids (Figure 10a and Figure A4). Notably, cardiac contents of saturated lipids, palmitate and stearate, were significantly increased in Tg-*SCD* hearts compared to non-transgenic B6 control hearts (Figure 10b,c). Cardiac palmitate levels of Tg-*SCD* mice were 1.63-fold higher than those of B6 controls, i.e., the cardiac palmitate content of Tg-*SCD* hearts was 4.6 μg/mg compared to 2.8 μg/mg in B6 controls (Figure 10b). Cardiac stearate content of Tg-*SCD* mice was 5.0 μg/mg, and cardiac stearate content of B6 controls was 2.9 μg/mg (Figure 10c).

In contrast to increased saturated lipids (palmitate and stearate), unsaturated lipids (palmitoleate and oleate) contents of Tg-*SCD* hearts were not significantly different from those of non-transgenic B6 controls (Figure 10d,e).

Thus, 4.9-fold elevated cardiac protein levels of SCD, which catalyzes the rate-limiting step of monounsaturated lipid synthesis, did not increase cardiac contents of monounsaturated lipids (palmitoleate and oleate) in Tg-*SCD* mice. Instead, SCD triggered accumulation of saturated lipids in the heart, e.g., palmitate and stearate. This effect could contribute to cardiac dysfunction, because saturated lipids are cardiotoxic and enhance cardiomyocyte death and heart failure [6,7,32,33].

### 2.11. Up-Regulation of the Heart-Failure-Promoting AT1 Receptor in Tg-SCD Mice

By generating membrane lipids, SCD is also essentially involved in membrane phospholipid biosynthesis. Membrane phospholipid biosynthesis is triggered by excessive lipids when the lipid accumulation exceeds the requirements of cardiac energy production [33].

Membrane bilayer synthesis is coupled with membrane protein synthesis and protein folding [34]. In view of the accumulation of increased saturated lipids in Tg-*SCD* hearts, we asked whether SCD also changed cardiac protein contents of a membrane-spanning and major heart-failure-promoting protein, i.e., the angiotensin II AT1 receptor, Agtr1.

**Figure 11:**
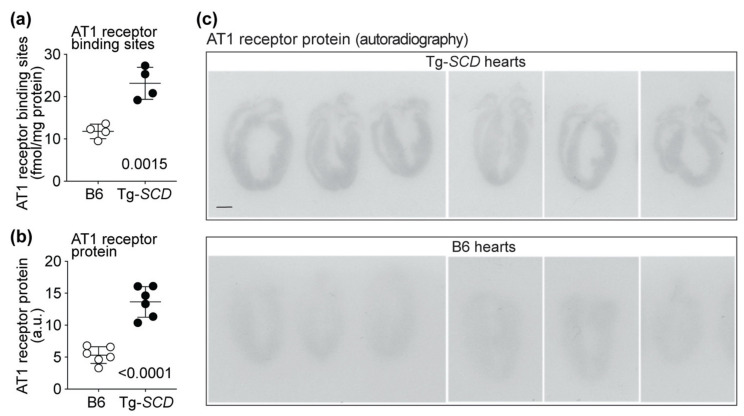
Up-regulation of the heart-failure-promoting AT1 receptor in Tg-*SCD* mice. (**a**) Number of AT1-receptor-specific binding sites on sarcolemmal membranes of 8-month-old, male Tg-*SCD* hearts and age-matched male B6 control hearts were determined by radioligand binding studies (±s.d., *n* = 4 mice/group, unpaired, two-tailed *t*-test); (**b**) increased cardiac AT1 receptor protein contents of Tg-*SCD* mice compared to B6 control mice were determined by autoradiographic imaging with AT1-receptor-specific antibodies (±s.d.; *n* = 6 mice per group; unpaired, two-tailed *t*-test); (**c**) autoradiographic images of AT1 receptor detection in Tg-*SCD* hearts and non-transgenic B6 control hearts by autoradiography with AT1-receptor-specific antibodies (*n* = 6 hearts per group; bar: 2 mm).

Radioligand-binding studies were performed with Sar^1^,[^125^I]Tyr^4^,Ile^8^-angiotensin-II to determine the number of AT1-receptor-specific binding sites on sarcolemmal membranes of Tg-*SCD* hearts compared to those of non-transgenic B6 hearts (Figure 11a). Radioligand-binding studies showed 1.97-fold-increased numbers of AT1-receptor-binding sites in Tg-*SCD* hearts compared to those in non-transgenic B6 hearts (Figure 11a).

Autoradiographic imaging of cardiac cryo-sections with AT1-receptor-specific antibodies revealed increased cardiac AT1 receptor protein contents of Tg-*SCD* hearts compared to those of non-transgenic B6 hearts (Figure 11b,c). The AT1 receptor protein content of Tg-*SCD* hearts was 2.57-fold higher than that of non-transgenic B6 hearts (Figure 11b,c).

**Figure A5:**
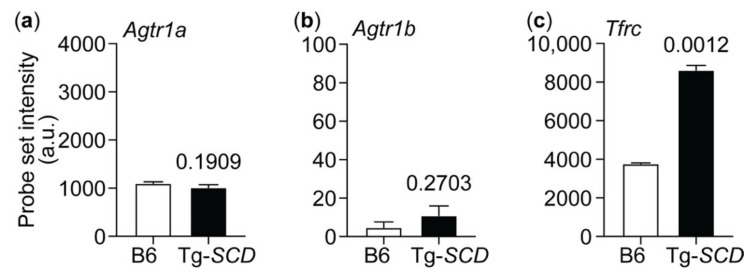
Expression levels of *Agtr1a*, *Agtr1b,* and *Tfrc* in Tg-*SCD* hearts. (**a**) Probe set intensities detecting *Agtr1a* (1436739_at) were not significantly different between Tg-*SCD* and non-transgenic B6 hearts; (**b**) intensities of probe set detecting *Agtr1b* (1446527_at) were below detection limit; (**c**) increased intensities of probe set detecting *Tfrc* (1452661_at) indicate up-regulation of *Tfrc* as an angiotensin-II-responsive and oxidative stress-induced gene. Data of two gene chips per group with cRNAs from four hearts per gene chip are shown (±s.d.; *n* = 2 gene chips per group; *p* values are indicated and were determined by the unpaired, two-tailed *t*-test).

AT1 receptor (*Agtr1*) expression levels were determined by microarray gene expression analysis. Probe set intensities of *Agtr1a* were not significantly different between Tg-*SCD* and non-transgenic B6 hearts (Figure A5a). *Agtr1a* is the major AT1 receptor in the murine heart, whereas the expression of *Agtr1b* was below the detection limit (Figure A5b). Thus, elevated cardiac *SCD* contents lead to increased cardiac AT1 receptor protein levels in Tg-*SCD* mice, whereas *Agtr1a* expression levels are not affected.

In agreement with increased functional AT1 receptor binding sites, angiotensin-II-responsive genes [35] were also up-regulated in Tg-*SCD* hearts, i.e., fibrosis-related genes, *Col1a2* and *Col3a1* (c.f. Figure 8e,f). In addition, the oxidative-stress-induced transferrin receptor 1, *Tfrc,* was elevated in Tg-*SCD* hearts (Figure A5c). The up-regulation of *Tfrc* could be a consequence of cardiac AT1-receptor-stimulated generation of reactive oxygen species [35,36].

**Figure A6:**
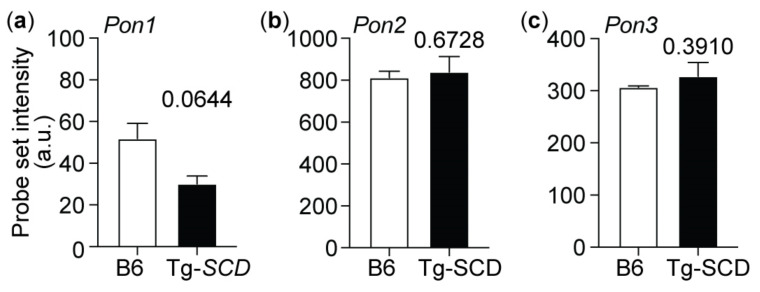
Cardiac expression levels of paraoxonase enzymes were not altered in Tg-*SCD* mice. (**a**) Intensities of probe set detecting *Pon1* (1418190_at) were below detection limit; (**b,c**) intensities of probe sets detecting *Pon2* (1450686_at) and *Pon3* (1419298_at) were not significantly altered in Tg-*SCD* hearts compared to non-transgenic B6 controls. Data of two gene chips per group with cRNAs from four hearts per gene chip are shown (±s.d.; *n* = 2 gene chips per group; *p* values are indicated and were determined by the unpaired, two-tailed *t*-test).

In contrast, expression levels of paraoxonase enzymes 1-3 (*Pon1, Pon2, Pon3*), which could detoxify oxidated lipids [37] and alleviate angiotensin-II-induced heart failure [38], were unaltered in Tg-*SCD* hearts (Figure A6a–c).

Taken together, accumulation of excessive cardiac lipids in Tg-*SCD* hearts is accompanied by increased protein levels of the heart-failure-promoting angiotensin II AT1 receptor and up-regulation of angiotensin-II-responsive genes.

### 2.12. SCD Expression Enhances the Number of Cell-Surface AT1-Receptor-Binding Sites of HEK Cells

**Figure 12:**
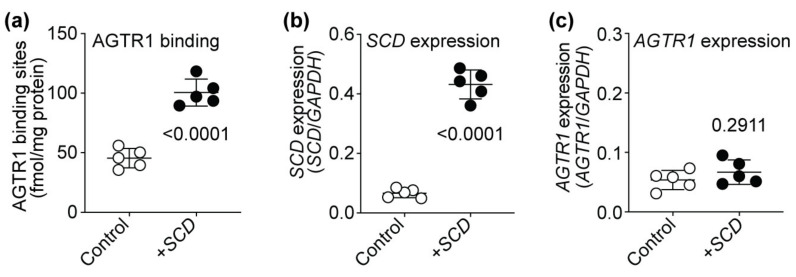
*SCD* expression enhances the number of cell-surface AT1-receptor-specific binding sites of HEK cells. (**a**) Number of AT1 receptor (AGTR1)-specific binding sites of HEK cells with stable AT1 receptor expression and transient transfection of *SCD* expression plasmid (+*SCD*) or control plasmid (Control); (**b**) quantitative real-time qRT-PCR determination of *SCD* expression levels of AT1-receptor-expressing HEK cells after transfection with *SCD* expression plasmid or control plasmid; (**c**) *SCD* did not affect *AGTR1* expression of HEK cells (±s.d., *n* = 5 biological replicates, unpaired, two-tailed *t*-test).

Does *SCD* alter AT1 receptor protein levels and the number of AT1 receptor binding sites in non-cardiomyocyte cells? We analyzed the impact of *SCD* on the number of AT1 receptor binding sites of AT1-receptor-expressing HEK (human embryonic kidney) cells to investigate whether *SCD* also affects the number of AT1 receptor binding sites of non-cardiomyocyte cells. HEK cells with stable AT1 receptor expression were transiently transfected with *SCD* expression plasmid or control vector, and the number of AT1 receptor binding sites was determined (Figure 12). Experiments found that increased *SCD* expression also significantly increased the number of AT1-receptor-specific binding sites of HEK cells (Figure 12a,b). Complementary to Tg-*SCD* hearts (cf. Figure A5), increased *SCD* expression did not affect the expression level of the AT1 receptor, *AGTR1,* in HEK cells (Figure 12c). Notably, expression levels of *AGTR1*, which was stably expressed in HEK cells under control of the ubiquitous CMV promoter, were not significantly different between HEK cells with and without *SCD* co-expression (Figure 12c). Together, these data show that *SCD* increases the number of AGTR1-specific binding sites in HEK cells and Tg-*SCD* hearts. In contrast, elevated *SCD* contents do not lead to increased expression levels of *AGTR1* in HEK cells and Tg-*SCD* hearts.

### 2.13. SCD Expression Increases AT1 Receptor-Cerulean Protein Levels of HEK Cells

**Figure 13:**
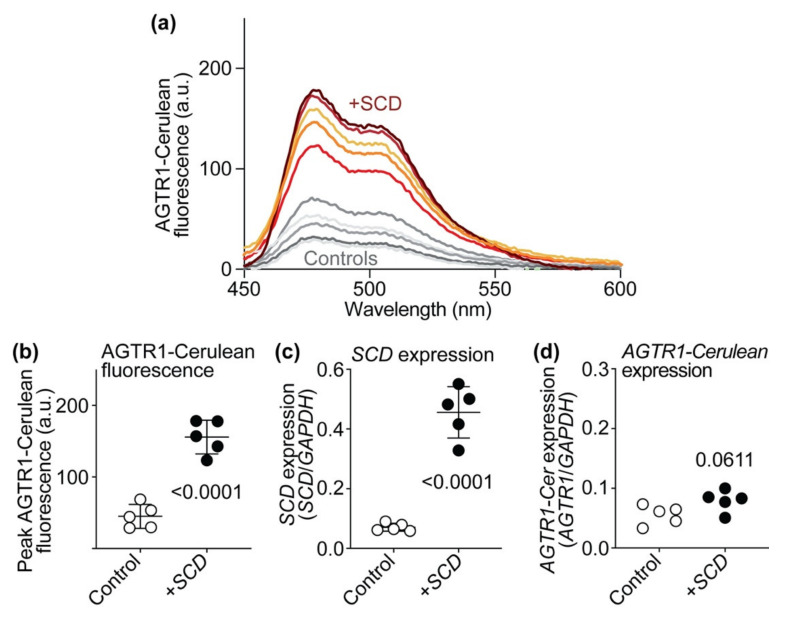
*SCD* expression increases AT1 receptor-Cerulean protein levels of HEK cells. (**a**) Fluorescence spectroscopic determination of AGTR1-Cerulean protein levels of HEK cells without (Controls) and with co-expression of *SCD* (+*SCD*); (**b**) quantitative data of AGTR1-Cerulean peak fluorescence levels at 475 nm without and with *SCD* co-expression; (**c**) *SCD* expression levels of HEK cells without and with *SCD* co-transfection were normalized to *GAPDH*; (**d**) *AGTR1-Cerulean* expression levels of HEK cells without and with *SCD* co-transfection (±s.d., *n* = 5 biological replicates, unpaired, two-tailed *t*-test).

The effect of *SCD* expression on protein levels of the AT1 receptor (AGTR1) was determined with an AT1 receptor with the C-terminally fused variant of the green fluorescent protein Cerulean, *AGTR1-Cerulean* (Figure 13).

The AGTR1-Cerulean was quantified by fluorescence spectroscopy. HEK cells were transiently transfected with *AGTR1-Cerulean*, and AGTR1-Cerulean fluorescence was quantified without and with co-expression of *SCD*. Fluorescence spectroscopy showed that expression of *SCD* increased protein levels of AT1 receptor-Cerulean (AGTR1-Cerulean), which was determined by fluorescence spectroscopy (Figure 13a,b).

As a control, *SCD* did not significantly alter the expression level of *AGTR1-Cerulean* (Figure 13c). Together, these experiments show that *SCD* also increases protein levels of AGTR1 and AGTR1-Cerulean in cultured human embryonic kidney cells.

## 3. Discussion

This study characterized the phenotype of Tg-*SCD* mice with myocardium-specific expression of *SCD* in the heart. The Tg-*SCD* mouse model recapitulates the up-regulated cardiac *SCD* levels of different experimental heart failure models [6,7] and of patients with heart failure [7]. The *SCD*-transgenic mouse model was generated because the functions of *SCD* and *SCD* up-regulation in the heart are not known. On one hand, *SCD* could be beneficial for cardiomyocyte survival because *SCD* counteracts saturated fatty acid-induced cellular apoptosis [12,13,14]. On the other hand, *SCD* could be detrimental because data from Scd1-knockout mice show that *Scd1* deficiency protects the heart against obesity-induced cardiac damage of ob/ob mice [15,16].

Phenotyping of Tg-*SCD* mice showed several lines of evidence, which prove that moderately (4.9-fold) increased cardiac *SCD* levels promote symptoms of heart failure at an age of 8 months. First, Tg-*SCD* mice have an increased mortality during the observation period of 10 months. The survival of Tg-*SCD* mice was reduced to 80.92% in male mice and 69.85% in female mice, respectively. Second, Tg-*SCD* mice develop cardiac hypertrophy with an increased heart-weight to body-weight ratio and predominant enlargement of the left cardiac ventricle. Left ventricular enlargement was documented by histological and echocardiographic analysis. Third, Tg-*SCD* mice develop cardiac dysfunction with a decreased left ventricular ejection fraction of 25.7 ± 2.9% and reduced fractional shortening of 10.2 ± 1.3%. These symptoms of heart failure were accompanied by up-regulation of heart-failure-promoting lipid genes, *Fasn* and *Scd1*, and heart failure markers, *Retn* and *Adipoq,* in Tg-*SCD* hearts [7,27,28]. In addition, up-regulation of extracellular matrix proteins, *Col1a2* and *Col3a1,* could reflect adverse fibrotic remodeling of Tg-*SCD* mice with symptoms of heart failure [30].

*SCD* could enhance the development of heart failure by the accumulation of saturated cardiac lipids. Lipid analysis showed 1.6-fold increased cardiac palmitate and 1.7-fold increased cardiac stearate contents of Tg-*SCD* mice compared to those of non-transgenic B6 mice. In contrast to saturated lipids, *SCD* overexpression in the heart did not significantly increase unsaturated cardiac lipids such as palmitoleate and oleate. Because saturated lipids are cardiotoxic [6,32,33], *SCD* could promote cardiac dysfunction and cardiomyocyte degeneration through induction of saturated lipid load. The increase in saturated lipids could be mediated by the major palmitate-synthesizing enzyme, *Fasn*, which was up-regulated in Tg-*SCD* hearts. These data complement previous studies with *Scd1*-deficient mice [23,24]. Notably deficiency of *Scd1* in mice led to a decreased accumulation of cardiotoxic free fatty acids, triglycerides and ceramide [23,24], and even down-regulated the lipid-generating enzyme *Fasn* [24].

SCD is a 9-delta desaturase, and major lipids generated by SCD are palmitoleate and oleate. Nevertheless, Tg-*SCD* hearts did not show increased unsaturated lipids, although saturated lipids were increased. This lipid profile of Tg-*SCD* hearts could be a consequence of increased oxidative stress, which is a major characteristic of heart failure [36,39]. In agreement with this notion, the oxidative stress-induced gene, *Tfrc*, as a marker of increased oxidative stress [35], was highly expressed in Tg-*SCD* hearts.

In addition to a direct cardiotoxic effect of accumulated saturated lipids, this study found that Tg-*SCD* mice had increased cardiac levels of the heart-failure-promoting angiotensin II AT1 receptor. Tg-*SCD* hearts had a higher number of AT1-receptor-specific binding sites and increased AT1 receptor protein levels as determined by autoradiographic imaging.

The up-regulated AT1 receptor could enhance the heart failure phenotype of Tg-*SCD* mice in several ways. The AT1 receptor could promote cardiomyocyte death by up-regulation of the pro-apoptotic p53 [40]. In agreement with this notion, Tg-*SCD* hearts had increased p53 levels. The enhanced AT1-receptor-dependent pro-apoptotic activity in Tg-*SCD* hearts could counteract antiapoptotic and cell-protective activities of *SCD* in vivo, which are documented for cardiomyocytes and other cells [12,13,14].

The increased AT1 receptor protein of Tg-*SCD* mice could also contribute to myocardial fibrosis, which is enhanced by angiotensin II AT1 receptor stimulation during the pathogenesis of heart failure [35,41]. In agreement with enhanced angiotensin-II-stimulated signaling, gene expression analysis found increased expression of angiotensin-II-responsive genes in Tg-*SCD* hearts. Notably, there was an increased expression of extracellular matrix genes, *Col1a2* and *Col3a1*, which could be induced by chronic angiotensin II stimulation [35]. Increased myocardial collagen accumulation is part of the adverse remodeling process of heart failure and contributes to myocardial stiffness and cardiac dysfunction [30].

The increased generation of reactive oxygen species, ROS, is another heart-failure-promoting factor triggered by angiotensin II AT1 receptor stimulation [36]. Up-regulation of the angiotensin-II-responsive and oxidative-stress-induced *Tfrc* [35] could reflect the exaggerated angiotensin-II-stimulated oxidative stress response of Tg-*SCD* mice. This *SCD*-dependent increase in AT1-receptor-stimulated generation of ROS could contribute to the observed accumulation of cardiotoxic saturated lipids in Tg-*SCD* hearts [42].

In concert with enhanced ROS generation, the angiotensin-II-generating ACE-AT1 receptor axis could directly enhance the accumulation of cardiotoxic lipids because angiotensin II AT1 receptor stimulation inhibits lipolysis and increases the activity of fatty acid synthase [43,44]. Thereby, the *SCD*-induced AT1 receptor could actively promote the accumulation of cardiotoxic lipids in Tg-*SCD* mice. Moreover, the AT1 receptor could impair the *SCD*-mediated increase in unsaturated lipids, most likely due to enhanced AT1-receptor-mediated ROS generation and lipid peroxidation [42].

*SCD* caused AT1 receptor (AGTR1) protein up-regulation not only in the heart but also in non-cardiomyocyte cells. *SCD* increased the number of AT1-receptor-specific binding sites of transfected HEK cells. In addition, *SCD* augmented AGTR1-Cerulean protein levels of transfected HEK cells. Up-regulation of the membrane-spanning AT1 receptor protein could be a consequence of enhanced membrane phospholipid biosynthesis because excessive lipid accumulation accounts for enhanced membrane biogenesis [33]. Ensuing expansion of the ER membrane could account for improved protein folding and increased biosynthesis of the membrane-spanning AT1 receptor [34]. In addition, by its delta-9 desaturase activity, SCD is an enhancer of membrane fluidity [45]. Thereby, SCD could further modulate and ameliorate membrane protein folding [46]. However, additional studies will have to delineate the exact mechanism underlying SCD-dependent AT1 receptor protein up-regulation.

This study identified the heart-failure-enhancing activity of *SCD* in mice. *SCD* could also contribute to heart failure in patients because cardiac SCD protein contents are increased in cardiac biopsy specimens of patients with heart failure [7]. SCD enhances cardiac damage by increasing cardiotoxic saturated lipids. Increased contents of saturated lipids with membrane phospholipid saturation correlate with diastolic dysfunction in patients with heart failure [33]. *SCD*-inducing factors include saturated fatty acids, cholesterol, carbohydrates, and insulin [12,47,48]. All of these factors are major players of diabetic cardiomyopathy. Therefore, *SCD* could play a major role in patients with diabetes, who are at increased risk of developing heart failure. In agreement with a role of *SCD* in human heart failure pathogenesis, increased circulating markers of enhanced stearoyl-CoA desaturase activity were associated with an elevated risk of heart failure development and all-cause mortality in human subjects [17,49].

**Figure 14:**
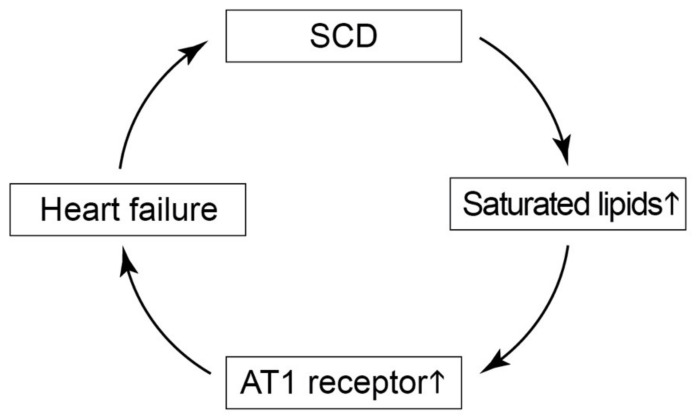
Scheme of *SCD*-induced pathomechanisms of heart failure. *SCD* triggers cardiotoxic saturated lipids and the heart-failure-promoting angiotensin II AT1 receptor. Saturated lipids, the AT1 receptor, and heart failure further augment the expression of *SCD* and thereby trigger a vicious circle of *SCD*-induced aggravation of heart failure.

*SCD* not only increased cardiotoxic saturated lipids but also augmented cardiac contents of the major heart-failure-promoting angiotensin II AT1 receptor. Pharmacological inhibition of AT1 receptor function by an ACE inhibitor, an AT1 receptor antagonist, or an ARNI (angiotensin receptor blocker and neprilysin inhibitor) is the mainstay of recommended prognosis-improving therapies of heart failure [4]. Treatment with an AT1 receptor blocker or ACE inhibitor was reported to down-regulate the increased expression of *SCD* [50]. Concomitantly, inhibition of the angiotensin II–ACE–AT1 receptor axis is expected to prevent detrimental AT1-receptor-stimulated lipid accumulation [43,44]. Consequently, AT1-receptor-blocking therapies are expected to interrupt the vicious circle of *SCD*-induced lipid load and AT1 receptor up-regulation during the pathogenesis of heart failure (Figure 14).

Because AT1 receptor levels are increased in diabetic cardiomyopathy [51], interruption of this vicious circle of *SCD*-induced AT1 receptor up-regulation by an AT1 receptor blocker is expected to be most efficacious in diabetic patients with heart failure. Future studies will have to investigate whether inhibition of *SCD* could synergize with an ACE inhibitor or AT1 receptor antagonist to prevent detrimental and heart-failure-enhancing activities of *SCD* in patients with *SCD*-mediated pathologies. A combination of an ACE inhibitor or AT1 receptor antagonist with an *SCD* inhibitor could also circumvent potential pro-atherogenic side effects of systemic *SCD* inhibition [52]. Those side effects could arise from the accumulation of lipid substrates of *SCD*, the saturated fatty acids (SFAs), which are pro-inflammatory and pro-atherogenic [53]. Pro-inflammatory and pro-atherogenic side effects of *SCD* inhibition in vivo can also be overcome by dietary fish oil supplementation, with a diet rich in omega-3 polyunsaturated fatty acids [54].

*SCD* inhibition in vivo is possible, and side effects of *SCD* inhibition do not seem to be a major concern, because in vivo data of an experimental *SCD* inhibitor showed beneficial therapeutic effects in an experimental model of liver injury [55]. Another *SCD* inhibitor showed beneficial therapeutic effects against diabetes and dyslipidemia [56]. This *SCD* inhibitor, MK-8245, is liver-targeted [56] and thereby overcomes side effects of systemic *SCD* inhibition such as skin barrier dysfunction and eye dryness [57]. *SCD*-induced cardiac dysfunction in Tg-*SCD* mice recapitulates the pathological effects of increased *SCD* levels in the heart. Therefore, treatment strategies should aim to target exaggerated and pathological *SCD* activities, while leaving physiological *SCD* effects untouched. With this strategy and in view of our study, the development of systemic *SCD* inhibitors for patient use appears as a promising strategy to improve heart failure treatment options.

## 4. Materials and Methods

### 4.1. GO Analysis of Whole-Genome Microarray Gene Expression Data of Experimental Heart Failure Models

In frame of this study, GO analyses of our cardiac whole-genome microarray gene expression data of experimental heart failure models were performed [6,7]. GO analyses were performed with the data of the following heart failure models: 6-month-old, male *Apoe-/-* mice with two months of AAC and age-matched, sham-operated, male *Apoe-/-* controls; 18-month-old, male *Apoe-/-* mice and age-matched, non-transgenic, male B6 controls; 8-month-old, male *Apoe-/-* mice with rosiglitazone treatment (30 mg/kg/d in drinking water) and age-matched, untreated, male *Apoe-/-* controls; 10-month-old, male B6 mice with 6 months of AAC and age-matched, sham-operated, non-transgenic, male B6 controls. Whole genome microarray gene expression data are available at the NCBI GEO database with the following accession numbers: GSE25765, GSE25766, GSE25767, and GSE25768. GO analyses were performed of GCOS/RMA processed data with Genespring GX Software (Agilent, Santa Clara, CA, USA). For GO analyses, probe sets with significantly different signal intensities were used (*p* ≤ 0.01; call present and/or signal intensity ≥100; ≥2-fold different signal intensity compared to respective control group). Probe sets with significantly different signal intensities compared to their respective control group were identified by TIGR MEV with the unpaired two-tailed *t*-test (just alpha).

### 4.2. Generation of Tg-SCD Mice and Animal Experiments

Tg-*SCD* mice were generated by the injection of *NotI*-linearized plasmid MyHC-SCD into the pronucleus of fertilized oocytes of B6 mice (2 ng/microL), as described [6]. For the generation of Tg-*SCD* mice, the cDNA encoding *SCD* was inserted into the *SalI*-*HindIII* sites of the plasmid MyHC [58]. The plasmid directs the expression of *SCD* under control of the myocardium-specific alpha-MHC promoter [58]. After DNA injection, 2-cell stage embryos were implanted into the oviducts of pseudopregnant foster mice. After weaning at an age of 3–4 weeks, offspring were subjected to genotyping PCR, and mice with stable genomic integration of the transgene were used for generation of C57BL/6-Tg(MHCSCD)Sjaa (Tg-*SCD*) mouse lines. The following DNA oligonucleotide primers were used for genotyping PCR of Tg-*SCD* mice: 5′-GGT TTC ACT TGG AGC TGT GGG TGA GG-3′; 5′-ATT AGG ACA AGG CTG GTG GGC ACT GGA GTG-3′. Oligonucleotides are specific for the transgenic *MHC-SCD* DNA and do not cross-react with the endogenous murine *Scd1* gene. The study used male and female Tg-*SCD* mice (C57BL/Tg(MHCSCD)2 Sjaa) at an age of 8 months. Controls were age-matched and sex-matched, male and female, non-transgenic C57BL/6 (B6) mice. Male and female mice were housed under SPF conditions in groups of 2–5 animals with a 12 h light cycle and had free access to food and water. Sperm of Tg-*SCD* mice are cryopreserved at Janvier Labs repository (C57BL/Tg(MHCSCD)2 Sjaa, No. 181.078 ETH Zurich). At an age of 8 months, mice were anesthetized i.p. with ketamine and medetomidine (75 and 0.5 mg/kg), and cardiac function parameters were assessed by echocardiography in the parasternal long-axis view by a Vivid 7 echocardiograph equipment (GE Healthcare GmbH, Solingen, Germany) with a 12 MHz linear array transducer [7]. Data were evaluated offline with the EchoPAC PC 3.0 Software (GE Healthcare GmbH, Solingen, Germany). The left ventricular cardiac ejection fraction (LVEF) was determined by the formula of Teichholz. For RNA isolation, protein, lipid, and histological analyses, animals were intracardially perfused with PBS under terminal anesthesia with ketamine and xylazine (200 mg/kg and 60 mg/kg) or euthanized. Hearts were rapidly isolated and dissected free of connective tissue. For RNA, protein and lipid isolation, heart specimens were immediately frozen in liquid nitrogen. For histological analysis, formalin-fixed, paraffin-embedded heart specimens were used. Animal experiments used a group size of 4–6. The number is based on the expected effect size and was determined in advance in frame of statistical pre-evaluation of the study by Novustat GmbH (Wollerau, Switzerland). The animal study and generation of transgenic mice were conducted according to NIH and Swiss guidelines and approved by the Cantonal Veterinary Office Zurich (ZH215/2020, date of approval 15.03.2021; and 145-G, date of approval 14.02.2013).

### 4.3. Antibodies

The following antibodies were used for the study: rabbit polyclonal anti-SCD-Scd1 antibodies (ab39969, knockout-validated, Abcam, Cambridge, UK), which were raised against a synthetic peptide; rabbit polyclonal anti-FASN antibodies, which were raised against an antigen encompassing amino acids 2205-2504 of human FASN [6]; rabbit monoclonal adiponectin antibody, which was raised against human adiponectin (C45B10 No. 2789, Cell Signaling Technology Inc., Danvers, MA, USA); affinity-purified, rabbit anti-AT1 receptor antibodies, which were raised against an antigen encompassing amino acids 306-359 of AGTR1 [59]; mouse monoclonal anti-p53 antibody (DO-1, sc-126, Santa Cruz Biotechnology Inc., Heidelberg, Germany); mouse monoclonal anti-Gnb antibody, which interacts with the N-terminus of Gnb2 (A-4, sc-166250, Santa Cruz Biotechnology Inc., Heidelberg, Germany); rabbit monoclonal anti-Gnb2 antibody (EP3262Y, ab108504, Abcam; Cambridge, UK); POD-conjugated AffiniPure F(ab)_2_ fragments of goat anti-rabbit IgG (Fcγ fragment specific with minimal cross-reaction to human serum proteins; Cat. No. 111-036-046, Jackson ImmunoResearch Europe Ltd., Ely, UK); POD-conjugated AffiniPure F(ab)_2_ fragment goat anti-mouse IgG (Fcγ fragment-specific, minimal cross-reactivity to human, bovine and horse proteins, Cat. No. 115-036-071, Jackson ImmunoResearch Europe Ltd., Ely, UK).

### 4.4. Histology and Immunohistology

After deparaffinization and rehydration, longitudinal cardiac paraffin sections of male and female Tg-*SCD* and non-transgenic B6 mice (age 8 months) were stained with hematoxylin and eosin (HE). For HE staining, sections were first incubated with Mayer’s hemalum solution (Sigma-Aldrich, Merck KGaA, Darmstadt, Germany) for 4 min, dipped in 0.1% HCl, and blued by rinsing steps with tap water. Thereafter, sections were stained with aqueous Eosin Y solution (0.5%; Sigma-Aldrich, Merck KGaA, Darmstadt, Germany) followed by a final washing step. Immunohistological analysis detected the cardiac SCD-Scd1 proteins on paraffin sections of 8-month-old, male and female Tg-*SCD* mice. The controls were age-matched, non-transgenic, male and female B6 mice. After deparaffinization and rehydration of paraffin sections, antigen retrieval was performed by microwave heating in antigen retrieval buffer (0.1 M citrate buffer, pH 6.0) for 20 min. After washing steps with PBS and inactivation of endogenous peroxidase activity by incubation with hydrogen peroxide solution (3%) for 5 min, blocking of unspecific binding sites was performed by incubation in blocking buffer (PBS with 3% BSA and 0.05% Tween-20) for 1 h at 37 °C. Cardiac sections were incubated with the primary antibody (knockout-validated, rabbit polyclonal anti-SCD-Scd1 antibodies; dilution 1:200; ab39969 Abcam) for 1 h at 37 °C. Unbound antibody was removed by washing with PBS-Tween, followed by a blocking step in blocking buffer and incubation for 1 h, at 37° with secondary POD-coupled anti-rabbit POD-conjugated AffiniPure F(ab)_2_ fragments of goat anti-rabbit IgG (Fcγ fragment specific with minimal cross-reaction to human serum proteins; Cat. No. 111-036-046, Jackson ImmunoResearch Europe Ltd., Ely, UK). After several washing steps with PBS, the substrate reaction was performed with a DAB (3,3′diaminobenzidine tetrahydrochloride)-enhanced liquid substrate system (Sigma-Aldrich, Merck KGaA, Darmstadt, Germany). The reaction was stopped by rinsing with water, and slides were mounted with Poly-Mount-Xylene (Polysciences Europe GmbH, Hirschberg, Germany). (Immuno-)histological sections were imaged with a DMI6000 microscope equipped with a DFC420 camera (Leica Microsystems GmbH, Wetzlar, Germany).

### 4.5. Immunoblot Detection of Proteins

For the detection of proteins by immunoblot, frozen hearts were pulverized under liquid nitrogen, and cardiac proteins were extracted by gentle agitation on ice with 500–750 μL of extraction buffer (10 mM Tris, pH 7.4, 1% sodium deoxycholate, 0.1% SDS, 5 mM EDTA, 1 mM beta-glycerophosphate disodium, 20 mM NaF, 1 mM sodium orthovanadate, 1 mM sodium molybdate, 1 mM PMSF, protease inhibitor cocktail 1:100). Insoluble material was pelleted by centrifugation (16,000× *g*, 4 °C, 10 min), and solubilized proteins were collected. Thereafter, proteins were concentrated and delipidated by addition of acetone/methanol (12:2, final concentration 83%) and incubation for 1 h at 4 °C. Precipitated proteins were collected by centrifugation and washed three times with 0.2 mL of ice-cold acetone. The pellet was solubilized in urea-containing, SDS sample buffer (supplemented with 2% SDS, 5% beta-mercaptoethanol and 6 M urea) for 90 min at room temperature. Proteins were separated by 7.5% or 10% SDS-PAGE under reducing conditions supplemented with urea (8 M) and transferred to PVDF membranes (Immobilon P, 0.45 microm; Millipore, Merck KGaA, Darmstadt, Germany) by semi-dry blotting (Trans-Blot^®^ SD Semi-Dry Electrophoretic Transfer Cell, Bio-Rad Laboratories Ltd., Hemel Hempstead, UK). After a blocking step with a blocking buffer (PBS with 0.2% Tween-20 and 5% BSA) for 1 h, the membrane was incubated with the primary antibody (dilution 1:2000) for 1 h at room temperature. Unbound antibody was removed by four washing steps of 5 min with washing buffer (PBS, 0.2% Tween-20), and the membrane was incubated for 30 min with the peroxidase-conjugated secondary antibody at a dilution of 1:40,000. After additional washing steps with PBS, bound POD-conjugated antibodies were visualized by chemiluminescence (Amersham ECL Prime Western Blotting Detection Reagent, or Amersham ECL Select, Cytiva Europe GmbH, Freiburg, Germany) and exposure of PVDF membranes to X-ray films.

### 4.6. RNA Isolation, Whole-Genome Microarray Gene Expression Profiling, and Real-Time qRT-PCR

Total RNA was isolated of HEK cells and hearts from 8-month-old, male Tg-*SCD* mice and age-matched, non-transgenic, male B6 controls by the RNeasy mini kit according to the instructions of the manufacturer (Qiagen GmbH, Hilden, Germany). Total RNA was processed for whole-genome microarray gene expression profiling with the Affymetrix One-Cycle cDNA Synthesis kit according to the Affymetrix protocol (Affymetrix GeneChip Expression Analysis Technical Manual, rev. 5, Affymetrix, Santa Clara, CA, USA), similarly as described [6,52,60]. Biotin-labeled, fragmented cRNA (15 microg per gene chip) was hybridized to GeneChip Mouse Genome MG430 2.0 arrays (Affymetrix, Santa Clara, CA, USA) with more than 45,000 probe sets. Signals of probe sets were processed with GCOS (version 1.4, Affymetrix, Santa Clara, CA, USA) to a target value of 300. Probe sets of Tg-*SCD* hearts with significantly different signal intensities compared to those of non-transgenic B6 controls (*p* ≤ 0.01; call present and/or signal intensity ≥100; ≥2-fold difference to B6 control group) were identified by TIGR MEV with the unpaired two-tailed *t*-test (just alpha). *SCD* and *AGTR1* expression levels of HEK cells were determined after reverse transcription by quantitative real-time qRT-PCR with LightCycler 480 SYBR Green I Master and a LightCycler 480 instrument (Roche Diagnostics AG, Rotkreuz, Switzerland) according to the protocol of the manufacturer. The following oligonucleotide primers were used for real-time qRT-PCR: AGTR1 forward 5′-CCG CCT TCG ACG CAC AAT GC-3′; AGTR1-reverse 5′-GGT CAG GCC CAG CCC TAT CG-3′; SCD-forward 5′-TTC GTT GCC ACT TTC TTG CG-3′; SCD-reverse 5′-AAG TTG ATG TGC CAG CGG TA-3′; GAPDH forward 5′-CAA ATT CCA TGG CAC CGT CAA G-3′; GAPDH reverse 5′-GGC CAT CCA CAG TCT TCT GG-3′. DNA oligonucleotides were purchased from Microsynth AG (Balgach, Switzerland). Microarray gene expression data of Tg-*SCD* mice are available at the NCBI GEO database with accession number GSE120020.

### 4.7. GC Analysis of Cardiac Lipids

Cardiac lipids were extracted by the method of Folch [61]. For lipid analysis, hearts of 8-month-old Tg-*SCD* mice and age-matched, non-transgenic B6 controls were rapidly frozen in liquid nitrogen. Frozen hearts were pulverized under liquid nitrogen with a pestle and mortar and extracted twice with 10 mL of chloroform/methanol (2:1) for 10 min followed by extraction with acidified chloroform/methanol (2:1). Lipid extracts were collected by centrifugation (620× *g*), and solvents were evaporated. The residual lipid extract was dissolved in 4 mL of chloroform/methanol (2:1). Hydrophilic contaminants were extracted with 50 mM CaCl_2_ (800 microL). The lipid phase was collected and subjected to the formation of fatty acid methyl esters (FAMEs) by transesterification with 3 N methanolic HCl. FAMEs were analyzed with a gas chromatograph (Focus, Thermo Scientific, Fisher Scientific AG, Reinach, Switzerland) equipped with a flame ionization detector and a DB-23 column (Agilent J&W; Agilent, Santa Clara, CA, USA). FAME reference standards (Supelco 37 component FAME mix, Sigma-Aldrich, Merck KGaA, Darmstadt, Germany) were used for identification, and an internal standard was included for quantitative lipid analysis.

### 4.8. Radioligand Binding Studies

Sarcolemmal angiotensin II AT1-receptor-specific binding sites were determined by radioligand binding. Radioligand binding studies were performed with sarcolemmal membranes, which were isolated from 8-month-old Tg-*SCD* mice and age-matched, non-transgenic B6 controls. Heart tissue was homogenized in a 10-fold volume of homogenization buffer (10 mM Tris, 1 mM EDTA, pH 7.4, supplemented with 300 mM sucrose and proteinase inhibitor cocktail) on ice with an Ultra-Turrax homogenizer (15,000 rpm). After centrifugation (1000× *g*, 10 min, 4 °C), the supernatant was centrifuged for 30 min at 40,000× *g* at 4 °C. The pellet was resuspended in buffer (0.6 M KCl, 30 mM histidine, pH 7.0) and centrifuged (40,000× *g*, 20 min, 4 °C). After the final centrifugation step, the pellet was resuspended in binding buffer (50 mM Tris, pH 7.4, supplemented with 10 mM MgCl_2_, 0.2% BSA and protease inhibitors) and stored at −80 °C. Radioligand binding was performed in triplicates in 100 μL of binding buffer with 100 μg of membrane protein for 60 min at 18 °C with Sar^1^,[^125^I]Tyr^4^,Ile^8^-angiotensin-II (specific activity 2200 Ci/mmol, Perkin Elmer Inc., Waltham, MA, USA). Non-specific binding was determined in the presence of the AT1-receptor-specific antagonist, losartan (1000-fold molar excess). After 60 min of incubation, the reaction was stopped by addition of ice-cold binding buffer (4 mL), followed by rapid filtration over glass fiber filters (Whatman GF/C) and three washing steps with binding buffer. Filter-bound radioactivity was quantified in a beta-counter by scintillation counting. For radioligand binding studies, adherent HEK cells were grown on 6-well plates. Before radioligand binding, HEK cells were starved for 3 h in DMEM with 0.2% FCS. Binding was performed with Sar^1^,[^125^I]Tyr^4^,Ile^8^-angiotensin-II in HEPES-buffered DMEM supplemented with protease inhibitor cocktail for 4 h, at 4 °C. Non-specific binding was determined in the presence of a 1000-fold molar excess of losartan. After three washing steps with ice-cold DMEM, cells were solubilized with 2 M NaOH, and radioactivity was determined by scintillation counting.

### 4.9. Imaging of Cardiac AT1 Receptors by Autoradiography

Autoradiographic imaging of cardiac AT1 receptors was performed by radio-immunohistochemistry with longitudinal heart cryosections prepared from Tg-*SCD* and non-transgenic B6 control mice (age: 8 months). Cryostat sections with 10 μm thickness were cut on a cryostat (Microm HM550), air-dried, and incubated in a blocking buffer (PBS supplemented with 5% BSA) for 1 h followed by incubation with affinity-purified anti-AGTR1 antibodies (dilution 1:200) from rabbit [59] for 2 h at room temperature. Unbound antibodies were removed by washing steps with buffer. After another blocking step with 10% goat serum, incubation with [^125^I]-labeled secondary antibodies (NEX155250UC, [^125^I]-labeled goat anti-rabbit IgG, ~3000 Ci/mmol; Perkin Elmer Inc., Waltham, MA, USA) diluted in blocking buffer was performed for 1 h at room temperature. Unbound antibodies were removed by several washing steps with PBS, and autoradiographic imaging of sections was performed by exposure to X-ray films.

### 4.10. Culture of HEK Cells and Fluorescence Spectroscopy

HEK293-AT1 cells with stable expression of *AGTR1* were cultured in a humidified atmosphere with 5% CO_2_ in DMEM (supplemented with 10% fetal bovine serum, 100 U/mL penicillin and 100 microg/mL streptomycin). HEK293 cells were transiently transfected with pcDNA3-based-expression plasmids encoding *SCD* and *AGTR1-Cerulean* by using Lipofectamine 2000 as a transfection agent. The *SCD*-pcDNA3 expression plasmid was generated by insertion of the *SCD*-cDNA into the *EcoRI*-*XhoI* sites of pcDNA3. The *AGTR1* and *AGTR1-Cerulean* expression plasmids were described previously [62]. Forty-eight hours after transfection, cells were starved for three hours in DMEM supplemented with 0.2% FCS and used for radioligand binding studies or cellular fluorescence measurements. For fluorescence measurements, *AGTR1-Cerulean*-expressing HEK cells were detached by short trypsinization (1–2 min) with 2 mL PBS-trypsin (PBS supplemented with 0.025% trypsin, 0.01% EDTA). Trypsin was rapidly removed by two washing steps with DMEM. After the second washing step, cells were suspended at a cell density of 1 × 10^6^ cells/mL in incubation buffer (140 mM NaCl, 5.4 mM KCl, 1 mM MgCl_2_, 1.8 mM CaCl_2_, 20 mM HEPES, pH 7.4). The cell suspension was transferred into a fluorescence cuvette, and fluorescence emission spectra (450 nm–600 nm) were recorded under constant stirring with a magnetic stirrer at an excitation wavelength of 420 nm with a fluorescence spectrometer (LS55, Perkin Elmer Inc., Waltham, MA, USA) and the following settings: excitation and emission slit width 10 nm; scan velocity 100 nm/min. The background fluorescence of HEK293 cells without *AGTR1-Cerulean* expression was subtracted. For data recording, the FL WinLab software was used.

### 4.11. Statistical Analyses

Experimental data were analyzed with GraphPad Prism 8. Comparisons between the two groups were made by the unpaired, two-tailed *t*-test. For comparisons between more than two groups, ANOVA with a post-test was used as indicated. *p*-values of < 0.05 were considered significant. Data are shown as means ± s.d.

## Data Availability

Whole-genome gene expression data are available at the NCBI GEO database with the following accession numbers: GSE120020, GSE25765, GSE25766, GSE25767, and GSE25768. All other data are shown in the manuscript and Supplementary Materials.

## Supplementary Materials

The following are available online at https://www.mdpi.com/article/10.3390/ijms22189883/s1, Tables S1–S4 show mouse genome microarray data (MG430 2.0 Array, Affymetrix) of four different heart failure models. Tables S1–S4 list probe sets with significantly different signal intensities compared to the respective control group (*p* < 0.01). Uncropped images of blots of Figure 6 and Figure 9 are shown as Supplementary Figure.
