# Supplementary figures and images for: Stearoyl-CoA Desaturase (SCD) Induces Cardiac Dysfunction with Cardiac Lipid Overload and Angiotensin II AT1 Receptor Protein Up-Regulation
Source: Int J Mol Sci. 2021 Sep 13;22(18):9883. doi: 10.3390/ijms22189883 (PMC8472087; doi:10.3390/ijms22189883)

# Blots Figure 6

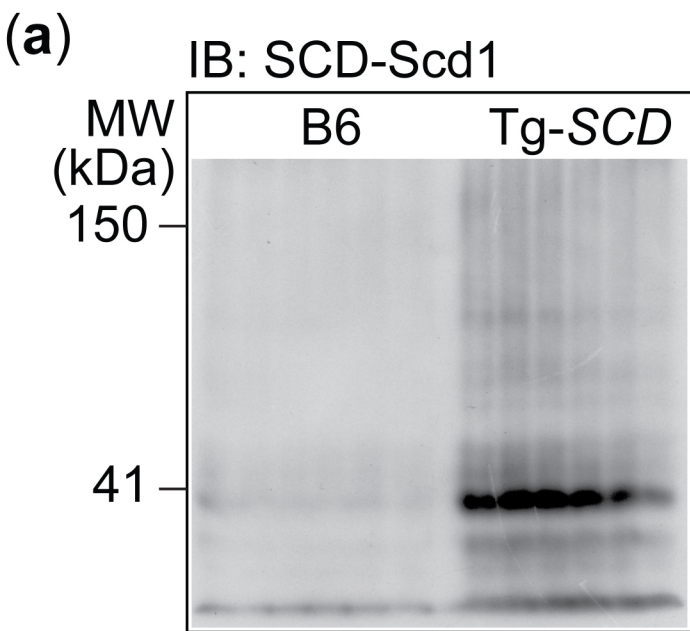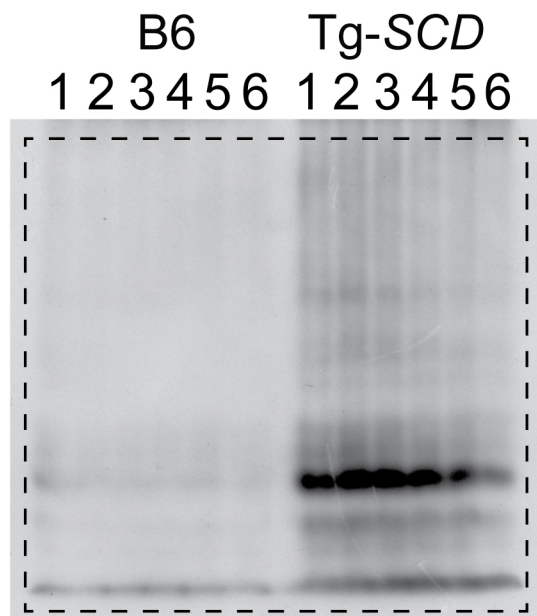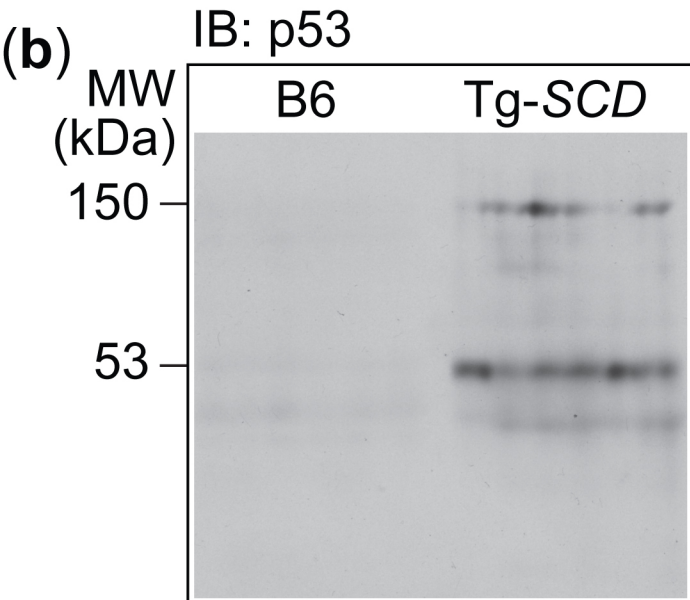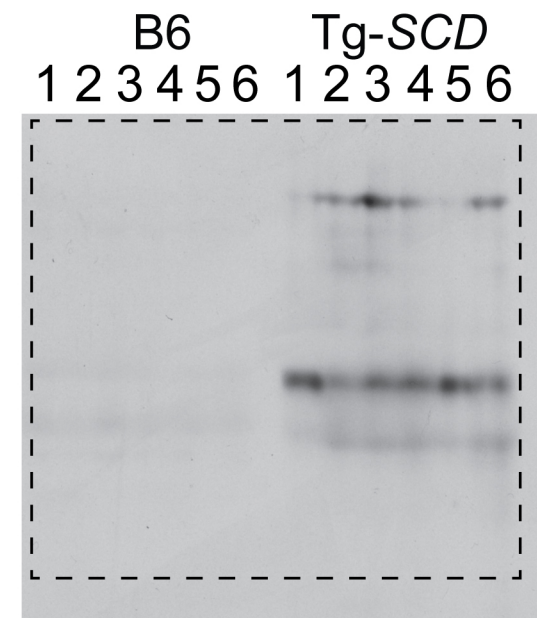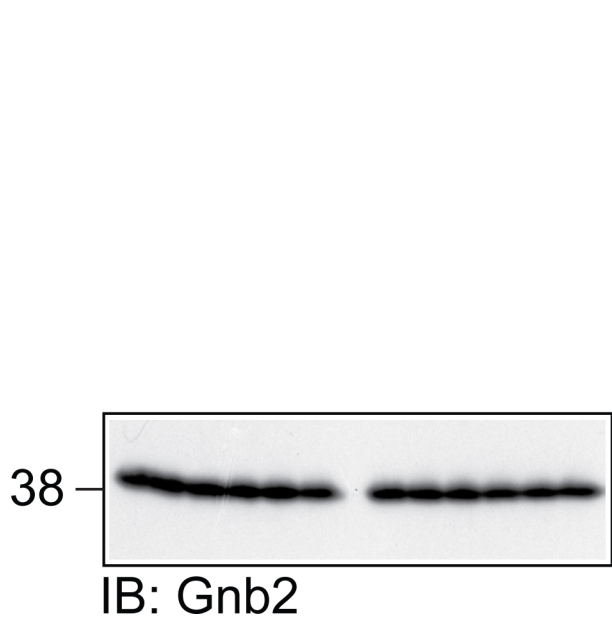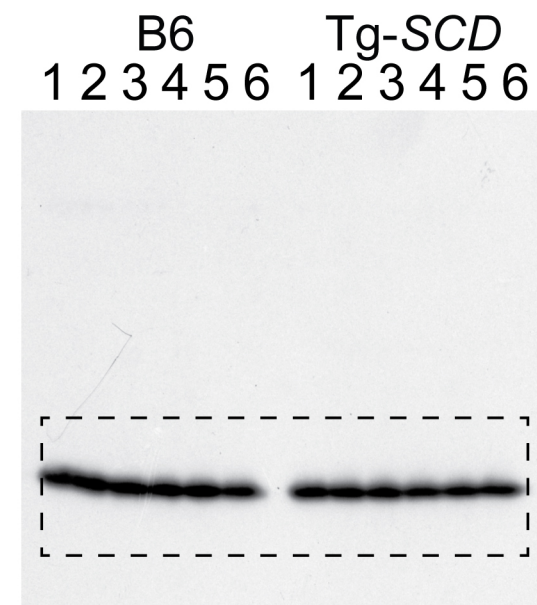

# Blots Figure 9

(a)

IB: Fasn

MW  
(kDa)  
250

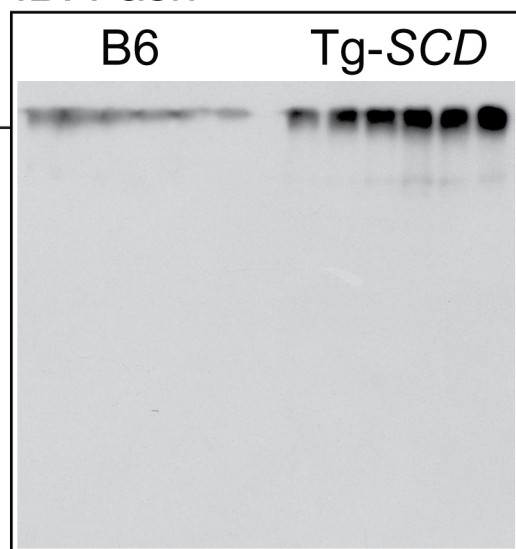

B6

Tg-SCD

1 2 3 4 5 6 1 2 3 4 5 6

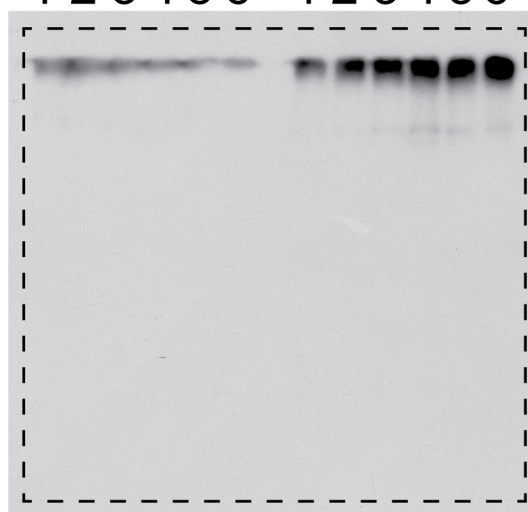

(b)

IB: Adipoq

MW  
(kDa)  
26

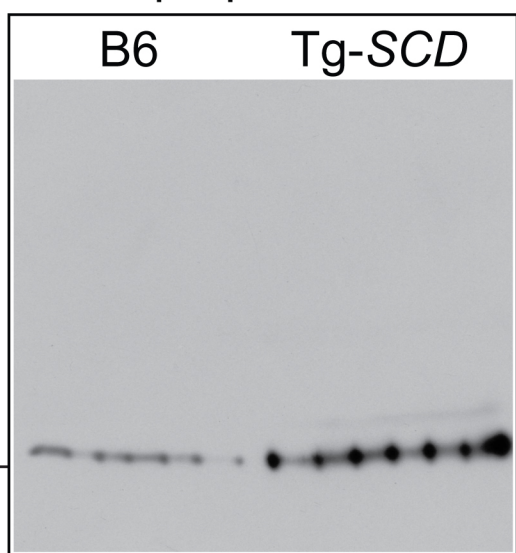

B6

Tg-SCD

1 2 3 4 5 6 1 2 3 4 5 6

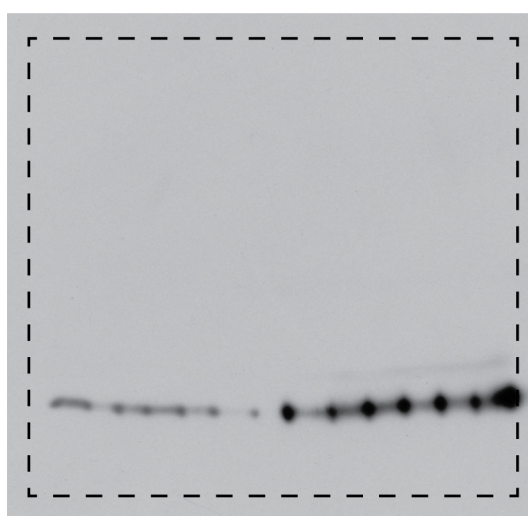

B6

Tg-SCD

1 2 3 4 5 6 1 2 3 4 5 6

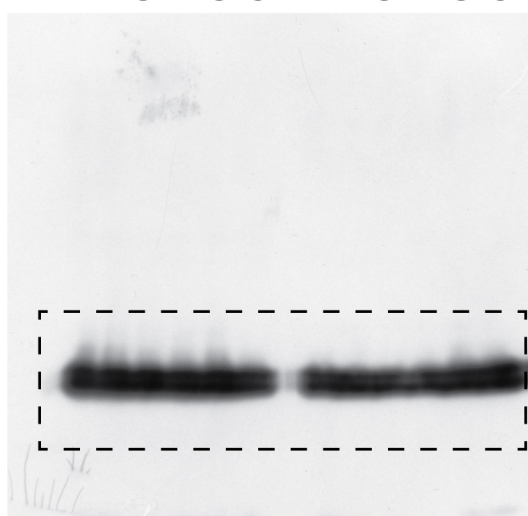

38

IB: Gnb

Supplement: Supplementary file 1 [file ijms-22-09883-s001.zip › Supplementary_Figure S1.pdf]
